# Monitoring Pathogens in Free-Living Large Herbivores in a Nature Reserve in the Netherlands

**DOI:** 10.1155/tbed/6948049

**Published:** 2025-08-08

**Authors:** Inês Marcelino, Jasmin Keizer, Gustavo Monti, Perry Cornelissen, Inge Santman-Berends, Jasper het Lam, Wim H. M. van der Poel

**Affiliations:** ^1^Infectious Disease Epidemiology (IDE), Wageningen University and Research (WUR), Wageningen, the Netherlands; ^2^Department Nature and Society, Staatsbosbeheer, Lelystad, the Netherlands; ^3^Institute for Biodiversity and Ecosystem Dynamics, University of Amsterdam (UvA), Amsterdam, the Netherlands; ^4^Department of Research and Development, Royal GD, Deventer, the Netherlands; ^5^Ruminant Health Department, Royal GD, Deventer, the Netherlands; ^6^Virology and Molecular Biology, Wageningen Bioveterinary Research (WBVR), Lelystad, the Netherlands

**Keywords:** Heck cattle, Konik horses, monitoring, nature reserve, prevalence, red deer, surveillance, the Netherlands

## Abstract

Monitoring and surveillance of pathogens are crucial for safeguarding animal and public health. While passive surveillance is more common for wild and free-living animals, active monitoring improves the detection and characterisation of specific pathogens relevant to animal and public health. In the *Oostvaardersplassen* (OVP) nature reserve in the Netherlands, an active monitoring system for Heck cattle (*Bos taurus*), Konik horses (*Equus caballus*) and red deer (*Cervus elaphus*) has been in place since 1997. This study utilised the data generated from the monitoring system to estimate pathogen prevalence and to evaluate the ongoing monitoring efforts. Yearly prevalences were calculated for each observed pathogen, and probability of freedom from infection was assessed for pathogens that were not detected. In cattle, the highest antibody prevalences (>35%–50%) were observed for Bovine herpesvirus 1 (BoHV-1), Bluetongue virus (BTV), Schmallenberg virus (SBV) and *Fasciola hepatica*, whereas lower (<25%) prevalences were detected for Bovine viral diarrhoea virus (BVDV), *Coxiella burnetii*, *Mycobacterium avium* subsp. *paratuberculosis* (*MAP*), *Salmonella* spp. and *S*. Dublin. Similar pathogens were observed in red deer and cattle, but prevalences were generally lower in red deer. In horses, Equine herpesvirus 1 and 4 (EHV-1 and 4), *Salmonella* spp., *S*. Dublin and *S*. Typhimurium were detected. Bovine leukaemia virus (BLV), *Brucella abortus*, *Mycobacterium bovis (bTB)* and *Leptospira interrogans* serovar Hardjo were not detected in cattle and red deer, nor were Equine infectious anaemia virus (EIAV), Equine influenza virus (EIV) and West Nile virus (WNV) observed in horses. Most of the detected pathogens are endemic in the Netherlands, while non-detected pathogens often have an official disease-free status. This study provides valuable insights into pathogen presence in free-living large herbivores at the OVP nature reserve. The current monitoring system is highly valuable and its effectiveness can be further enhanced through improvements, such as increased sampling efforts and pathogen prioritisation. This knowledge could guide the implementation of similar monitoring strategies in nature reserves across Europe.

## 1. Introduction

Animal health monitoring and surveillance systems are crucial to safeguard animal and public health. While monitoring involves the systematic collection and interpretation of animal health data without a predefined action plan, surveillance is more intensive, employing a defined strategy to address disease risks [1, 2]. In the European Union (EU), these systems are regulated under frameworks, such as Regulation (EU) 2016/429 and Regulation (EU) 2018/1882, focusing on diseases that pose a risk to animal health, as well as public health, potentially leading to significant economic losses and impacting animal welfare [3, 4]. When a disease is present, surveillance measures prevalence, identifies cases and tracks changes in disease patterns over time. In the absence of disease, it demonstrates freedom from disease, which is fundamental for international trade [2, 5]. Ultimately, the outcomes from monitoring and surveillance systems inform strategies for managing animal health and welfare.

Surveillance has been focused on livestock due to their economic importance and proximity to human populations [6, 7]. However, surveillance of wildlife is increasingly recognised as essential for monitoring both animal and human health, particularly in the context of emerging zoonotic diseases [8–10]. This reflects the interconnectedness of animal and human health, a key aspect of the One Health approach [11]. Therefore, it is essential to consider its importance for nature conservation. Wildlife, such as red deer (*Cervus elaphus*) or European bison (*Bison bonasus*), and domestic species, such as cattle (*Bos taurus*) and horses (*Equus ferus caballus*), are increasingly being introduced in nature reserves (referred in this study as free-living large herbivores), with the aim of contributing to a better functioning of ecosystems and providing opportunities to increase and maintain biodiversity [12, 13].

Despite their ecological importance, monitoring and surveillance systems for wild and free-living animals remain less developed than those for livestock. While livestock farming may pose risks to wild or free-living herbivore populations, wild herbivores can also threaten domestic herbivores, as both can often be infected by the same pathogens [9, 10]. Wildlife monitoring and surveillance rely on both passive and active methods, with passive surveillance being more common. However, inconsistent strategies (e.g., balancing both surveillance methods), diagnostic limitations and inadequate funding create practical and logistical challenges that might result in incomplete data and hinder detection and prevention of emerging threats [14–17]. To address these issues, standardising methodologies and centralising information are recommended to strengthen national systems [14–17]. Furthermore, inter and transdisciplinary collaboration is essential. The One Health approach provides a framework to integrate surveillance efforts across various animal populations, thereby improving coverage and effectiveness of surveillances systems [15–17].

In Europe, wildlife disease monitoring and surveillance systems vary by country, but are often integrated into overall animal health systems [15–18]. In the Netherlands, wildlife health monitoring programmes are coordinated by several institutes, such as the Dutch Wildlife Health Centre (DWHC), together with Utrecht University, National Institute for Public Health and the Environment (RIVM), Wageningen Bioveterinary Research (WBVR), Erasmus Medical Centre (Erasmus MC), Dutch Animal Health Services (Royal GD) and the Dutch Food and Consumer Product Safety Authority (NVWA). However, there is no official active monitoring programme specifically for large herbivores in nature areas, such as red deer [18]. To the authors' best knowledge, a notable exception is the *Oostvaardersplassen* (OVP) nature reserve, where an active animal health monitoring programme has been conducted since 1997 in close collaboration with Royal GD and WBVR.

In this context, this study aims to evaluate the frequency of health outcomes derived from the existing animal health monitoring programme for the free-living large herbivores at the OVP. Using data from this monitoring system, pathogen prevalence and the probability of freedom from infection were estimated in these animal populations. By assessing pathogen prevalence, evaluating the effectiveness of current monitoring and identifying potential areas for improvement, this study contributes to the broader goal of enhancing animal health monitoring in nature reserves.

## 2. Materials and Methods

### 2.1. Study Area and Animal Population

**Figure 1:**
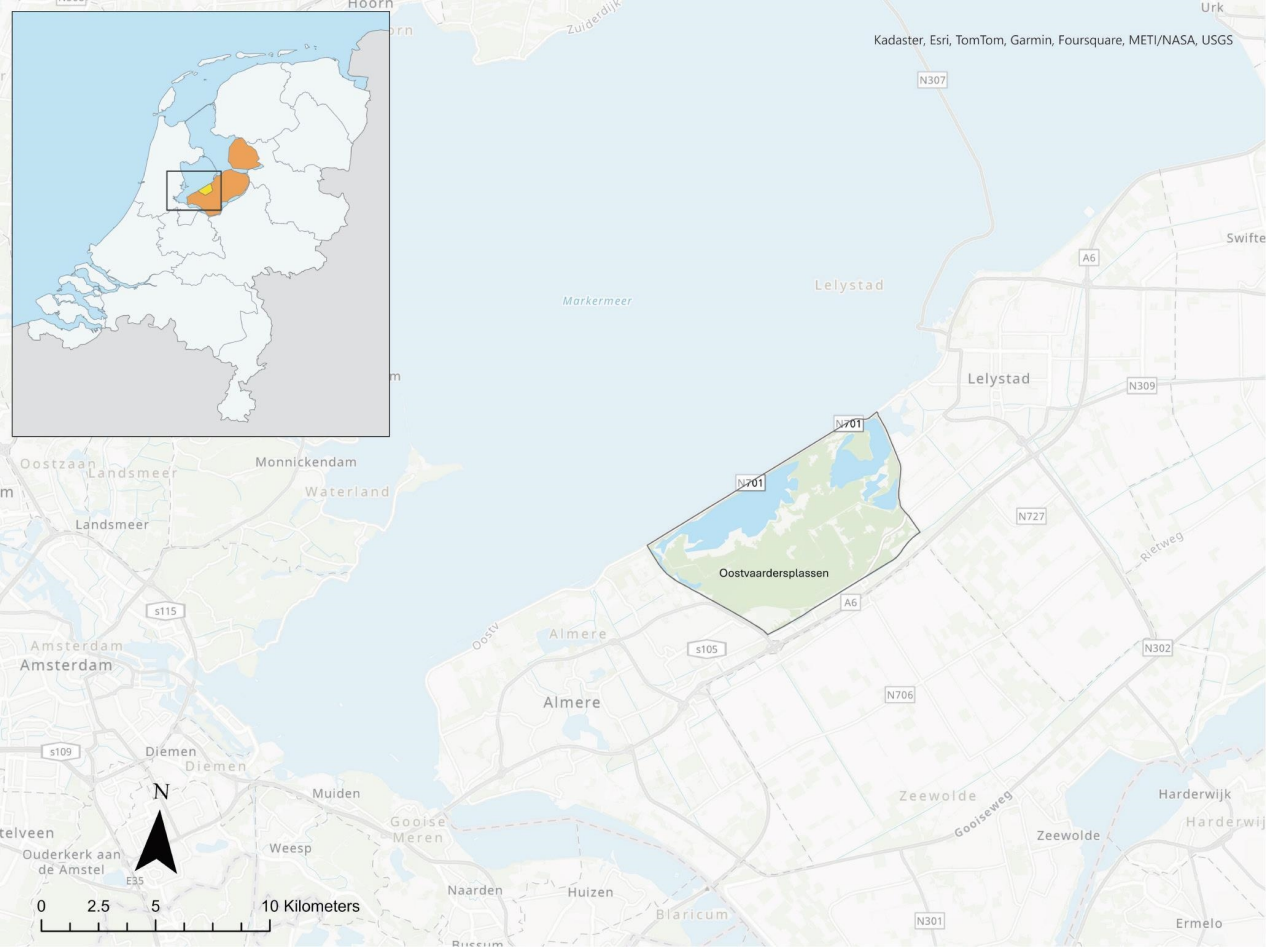
Location of the *Oostvaardersplassen* (OVP) nature reserve within the Netherlands. Orange denotes the province of Flevoland and yellow corresponds to the OVP. Map created using ArcGIS Pro.

The current study was conducted at the nature reserve OVP, which is part of the National Park Nieuw Land in the Netherlands (Figure 1). The OVP is a eutrophic wetland of 55 km^2^, consisting of a marsh (36 km^2^) and a dry border (19 km^2^) zone. The border zone consists mainly of non-inundated grasslands, along with grasslands that are inundated during winter (December to March), all of which are connected to surrounding forest. Three large herbivore species inhabit the area: Heck cattle, Konik horses and red deer. In 1983, 34 Heck cattle from Germany, Belgium and Switzerland were introduced in the area. Before introduction, these animals were examined for *Mycobacterium bovis* (bTB) by tuberculin skin test (TST), *Brucella abortus* by serum agglutination test (SAT) and complement fixation test (CFT), Bovine leukaemia virus (BLV) by agar gel immunodiffusion test (AGIDT) and *Mycobacterium avium* subsp. *paratuberculosis* (*MAP*) by CFT. In addition, foot and mouth disease vaccination was administered, as well as treatment against ectoparasites. Specific details on treatment were not recorded. In 1987 and 1989, 30 additional Heck cattle were introduced from Slikken van Flakkee, the Netherlands. In 1984, 18 Konik horses were introduced from Poland, followed by an additional 25 horses from the same country between 1986 and 1995. In 1992, 52 red deer were introduced from Scotland, Germany and the Netherlands. Five more red deer were introduced from former Czechoslovakia in 1993. Horses and red deer were equally tested according to import regulations, but data regarding testing was not available.

Management of the nature reserve considers grazing by large herbivores as an essential natural process for creating a diverse and suitable environment for wetland bird species. In the past, the large herbivore populations were controlled by feed availability and competition and relative severity of the different winters in the Netherlands. The area is fenced and large predators are not present. In 2018, a provincial policy plan was created and implemented in the management of the OVP to increase biodiversity and enhance animal welfare [19]. Herbivore population size is since controlled through active culling to decrease grazing pressure. No other interventions are implemented, except for the removal of injured animals due to welfare reasons. Simultaneously, the management aims to guarantee suitable animal welfare, with emphasis on the physical health and social behaviour of the large herbivores.

### 2.2. Animal Sampling and Data Collection

Since the 1990s, a monitoring system has been in place to assess animal health, aiming to prevent diseases that affect both public health and animal husbandry while ensuring a healthy animal population. The system involves regular inspections by the Dutch Forestry Service (*Staatsbosbeheer* [SBB]) rangers alongside a veterinarian. Initially developed for cattle health, the system later expanded to include horses and red deer. In its early years (mid to late 1990s), monitoring consisted of biannual faecal sampling from cattle and horses to test for a limited set of pathogens. Over time, the protocol was updated to improve surveillance and establish clear monitoring goals for the OVP [20]. In 1997, it was established that the cattle population was required to be free of foot-and-mouth disease virus (FMDV), rabies, anthrax, *Brucella abortus*, BLV, bTB and Bovine spongiform encephalopathy (BSE), with diseases categorised as shown in Table S1. The monitoring system has been periodically re-evaluated, leading to improved red deer and horse sampling schemes and additional testing, such as vector-borne and horse-specific pathogens [21–23].

The number of animals to sample in the monitoring system was statistically estimated to determine presence/absence of disease, based on population size, design prevalence (DP), and an assumption of 100% test sensitivity (Se) and specificity (Sp), using the following formula:

(1) $$\begin{array}{c} \begin{array}{c} n=\left(1-{\left(1-p\right)}^{\frac{1}{d}}\right)\times \left(N-\frac{d}{2}\right)+1 \end{array}, \end{array}$$

where *n* is sample size, *p* the probability of finding at least one positive case, *d* the minimum expected number of affected animals in the population and *N* the population size [2]. To detect at least one infected animal in a herd of 300–500 animals, with a determined DP of 25%, it was estimated that a minimum of 11 animals per species per year is required. The goal is to obtain a similar number of samples from males and females annually. As determined by the veterinary committee, the annual aim is to complete at least 33 necropsies, in which 33 blood samples are taken, with an additional 22 blood samples (11 from cattle and 11 from red deer) when feasible.

Blood and faecal samples for pathogen testing were collected by the rangers after shooting, and shot animals were submitted for pathological examination (results were not analysed as they were outside the scope of this study). Samples were collected throughout the year, but while random sampling was pursued, it was not always feasible. Instead, rangers aimed to select a healthy-looking individual from a different group each time, making the sampling method purposive. Additionally, samples obtained by convenience from animals culled for population control were sometimes included. Shot animals and post-mortem blood samples were transported to the Dutch Animal Health Services (Royal GD) facilities for pathological examination and pathogen testing of various bacteria, viruses and parasites (Table 1), following the protocol established by the veterinary committee and the Dutch national regulations for these animal species. All the diagnostic testing was performed according to standard protocols, either at Royal GD or WBVR laboratories. Serology, molecular and direct methods were used for detection of viruses and bacteria. The different techniques applied in detection of parasites were serology (for *F. hepatica*), Baermann technique (for lungworm detection), McMaster technique (in all other endoparasites) and external inspection (for ectoparasites).

### 2.3. Data Analysis

#### 2.3.1. Seroprevalence and Prevalence Calculations

A retrospective longitudinal cross-sectional study was conducted to assess pathogen seroprevalence and prevalence, utilising test results obtained through the existing monitoring system at the OVP between the years 1997 and 2023. Given that the number of available test results varied between years, only pathogens with at least three test results per year were included in the prevalence calculations for that year. All data handling and analyses were performed in R (version 4.4.1) [24], using RStudio (version 2024.04.2+764), and data manipulation was done using the R package *tidyverse* [25].

Yearly apparent prevalence (AP) was estimated as point prevalence for each species and per pathogen. For each year *t*, AP was calculated as the number of animals with a positive test result *x*(*t*) divided by the total number of animals tested *n*(*t*). The 95% confidence intervals (CIs) for AP were estimated by the Wilson's method [26] using the function *epi.prev* from the R package *epiR* [27]. True prevalence (TP) was then estimated from AP using a Bayesian framework that accounted for imperfect diagnostic test performance [28]. A non-informative beta (1,1) prior distribution was assumed for TP, and external Se and Sp values were fixed. Two chains of 50,000 iterations were simulated, of which the first 10,000 were discarded as burn-in. Model convergence was assessed using the multivariate potential scale reduction factor [29]. Posterior distributions were summarised using the median and the 95% uncertainty interval (UI). Models were implemented using the function *truePrev* from the R package *prevalence* [30].

Se and Sp values for each test used were obtained from the institutions involved in the pathogen testing. When this information was unavailable, the values were obtained either from the Manual of Diagnostic Tests and Vaccines for Terrestrial Animals (Terrestrial Manual) of the World Organisation for Animal Health (WOAH) [31] or from literature search via PubMed and Scopus. The Se and Sp values per method and per pathogen with respective references are summarised in Table S2. For most pathogens, serological tests were used to estimate prevalence, except for *B. abortus*, bTB, *MAP*, *Salmonella* spp. and EHV-1 and 4, for which serological, molecular or other direct tests were used (and interpreted either in parallel or serially). When multiple diagnostic tests were used in parallel or serial combinations to detect a pathogen, the overall diagnostic performance was estimated under the assumption of conditional independence of tests [2]. For these calculations the functions *se.parallel*, *sp.parallel*, *se.series*, *sp.series* from R package *RSurveillance* were used [32]. When distinct individuals were tested for the same pathogen using different diagnostic tests within the same year, both estimates (AP/TP) and respective CIs were weighted according to sample size. The trend of the prevalence over the study period for each pathogen was visualised in plots using the R package *ggplot2* [33].

As the test results were not expressed in the same way for all parasites, for most except *F. hepatica*, results were expressed in presence/absence and/or parasitic load measured in eggs/oocysts per gram of faeces (EPG/OPG). *Eimeria* spp. results were presented qualitatively, categorised as ‘few' (up to 500 OPG) and ‘a lot' (between 500 and 5000 OPG). Faecal egg counts (FECs) were classified based on EPG values: low (<200 EPG) and high (>200 EPG) [34, 35].

#### 2.3.2. Probability of Freedom From Infection

As the aim of the monitoring was to detect presence/absence of pathogens, the probability of freedom from disease was assessed using the number of samples tested for each pathogen within each species population. When all samples from a given year tested negative, the probability of freedom from infection for that year was estimated based on Cameron and Baldock (1998) [36]. This analysis compares the null (*H*_0_) and alternative (*H*_a_) hypotheses based on the survey observations and diagnostic tests used. *H*_0_ is defined as the prevalence being equal to or greater than the DP specified by the user, while *H*_a_ is defined as prevalence being lower than the DP. The method accounts for a finite population and imperfect diagnostic tests with known Se and Sp. To determine whether a population is free from infection, the probability of the H_0_ should be low and the probability of *H*_a_ should be high, considering the user-defined Type I (*α*) and Type II (*β*) errors. The confidence of freedom from infection is calculated as: 1 − probability of *H*_0_. If both probabilities are high, the evidence is insufficient to conclude freedom from infection based on sample size used. For these estimations, an individually created R function *PFFI* was used [37]. The following input information was applied for a given year (*j*): *N*(*j*) = total population, *n*(*j*) = number of sampled individuals, *x*(*j*) = number of positive individuals, *p* = DP (25%), Se and Sp of the single or combined diagnostic tests used, *α* = Type I error (0.05) and *β* = Type II error (0.05). If different tests were used within the same year, the estimates were adjusted by sampling weights.

Additionally, sample sizes were assessed for lower DPs (5% and 10%) while considering both perfect and imperfect diagnostic test performances. For perfect tests, Formula (1) was applied. For imperfect tests, sample sizes were estimated using the online tool *Epitools FreeCalc*, which considers Se and Sp values for the diagnostic test used and uses a modified hypergeometric exact calculation method for small populations [38]. Estimations were based on the target population size of the nature reserve (300 cattle, 300 horses and 500 red deer) and the most commonly used diagnostic test for each pathogen.

## 3. Results

### 3.1. General Outcomes and Study Population Description

Heck cattle, red deer and Konik horses were tested for 36 pathogens (Table 1). Cattle were tested for 25 pathogens, red deer for 23 and horses for 17 pathogens. This number of pathogens tested reflected the testing protocol of the veterinary committee and the Dutch national regulations for these species. Of the pathogens tested, 17 pathogens were detected in cattle, 12 in red deer and nine in horses.

**Figure 2:**
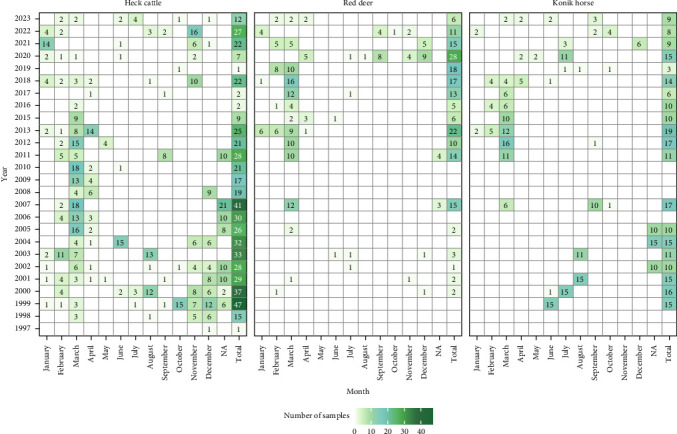
Number of animals sampled with either blood and/or faeces and/or from necropsy at the *Oostvaardersplassen* from 1997 to 2023 distributed by month. The classification NA represents samples that did not have a known date of collection apart from the year of collection.

Figure 2 provides an overview of the number of animals sampled (either blood and/or faeces and/or from necropsy) collected over time (1997–2023). For Heck cattle, prevalence and freedom from infection estimation results were included between the years of 1997 and 2023, for red deer from 2000 to 2023 and horses were included between 2007 and 2023. Horses samples collected between 1999 and 2005 were only examined for parasites in faeces.

Cattle sampling was the most consistent, with only six out of 26 years having fewer than 11 samples collected. It is worth noting that this figure refers to samples collected, not samples tested. Since, not all collected samples were suitable for testing, the actual number of samples available for each pathogen may vary even further (Tables S3–S8). Over the study period, a total of 984 animals were sampled. Of these, 56.3% were cattle, 19.3% were red deer and 24.4% were horses (Table 2). There were overall differences in the proportions of sex and age groups tested among the species, with a considerable proportion of samples with unknown sex and age (Table 2). The majority of cattle and deer samples were from females, while the proportion of sexes in horse samples was more balanced. Similarly, for age groups, samples from horses were collected rather consistently from all ages, while in cattle a lower number of samples from very young and senior animals was collected, and in deer, mostly young animals were submitted.

### 3.2. Pathogen Prevalence by Species

#### 3.2.1. Heck Cattle

**Figure 3:**
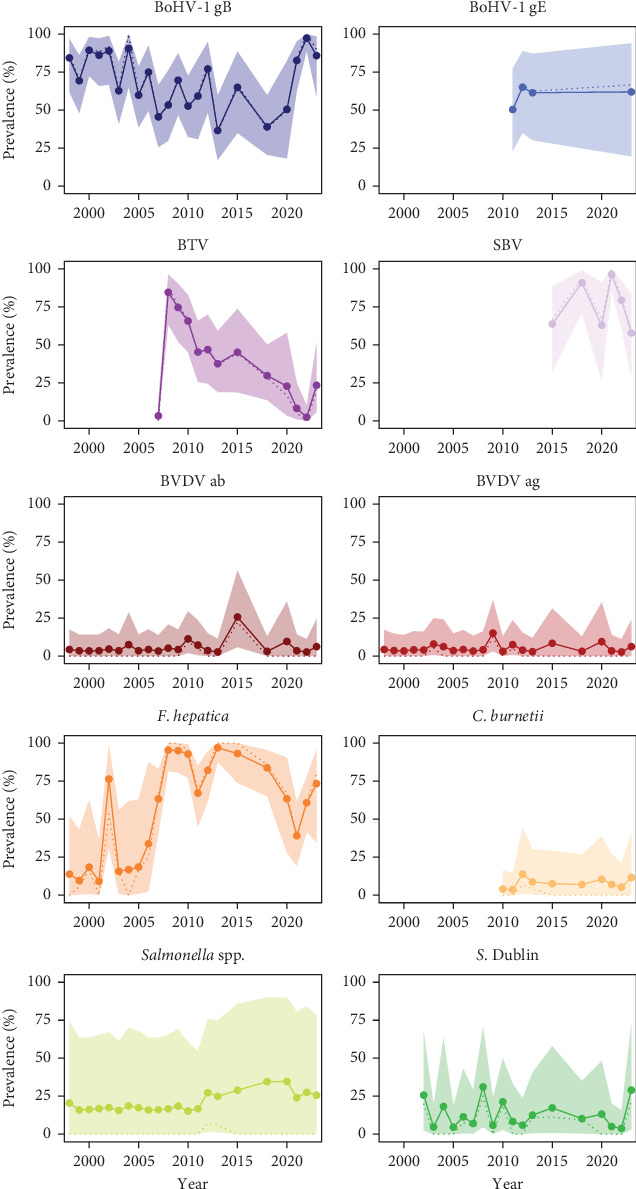
Apparent prevalence (AP, dotted line) and median true prevalence (TP, solid line) of detected pathogens in Heck cattle at the *Oostvaardersplassen*. The figure shows AP and TP in percentages between the years of 1999 and 2023. The shadow represents 95% uncertainty interval (UI) for TP. Each point represents a sampled year.

The study revealed the presence of several pathogens. Heck cattle tested positive for several different viruses, including BoHV-1 (for both glycoproteins gB and gE), BVDV (both antibodies [ab] and antigen [ag]), Bluetongue virus (BTV) and Schmallenberg virus (SBV). The only parasite measured with serological methods and detected during the study period was *F. hepatica*. Last, bacteria observed in cattle were *C. burnetii*, *Salmonella* spp. and *S*. Dublin. *MAP* was detected and is present; however, due to inconsistent testing protocol, TP was not inferred. When testing for *Salmonella* spp. resulted in a positive result, the sample was further identified to the species level. In cattle, one positive result was identified as *S*. Typhimurium and the other case was unidentified.  Figure 3 displays the AP and TP with their 95% UIs, between 1998 and 2023, for the different pathogens detected. Furthermore, all the detailed values for AP, TP and corresponding CIs and UIs can be consulted in Table S3. Last, cattle were tested for BSE, in accordance with international and national regulations, and no cases were identified.

Several parasitic pathogens, measured as EPG, were detected in all three animal species during the study period.  Table 3 presents the results for Heck cattle, with *Eimeria* spp., *S. papillosus* and Trichostrongylus/Strongylus eggs being the most frequently identified. *Moniezia expansa* was detected in only four cases, while *Eimeria* spp. was generally observed at ≤500 OPG (category ‘few'). Observed lungworm was identified as *Dictyocaulus viviparus*, and *Capillaria* sp. was detected when investigating other parasites. Median EPG for *Nematodirus* spp., Trichostrongylus/Strongylus eggs and *S. papillosus* per year was ≤200. Table S6 provides the number of samples, proportion of positive results per year and median EPG values.

#### 3.2.2. Red Deer

**Figure 4:**
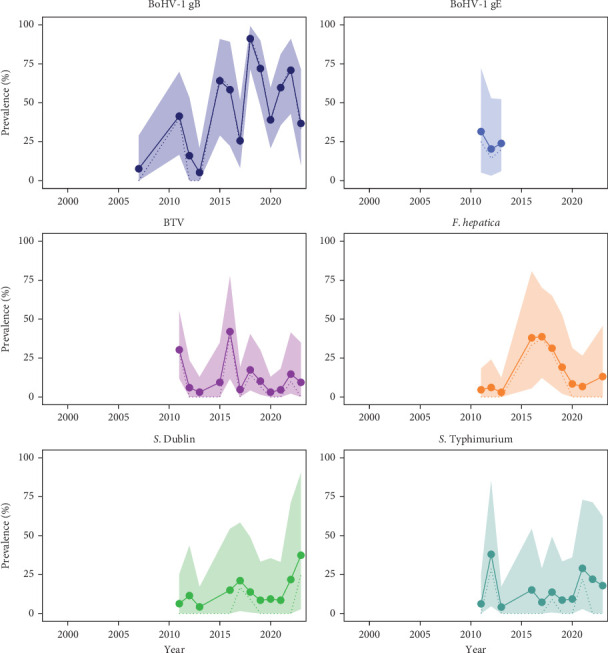
Apparent prevalence (AP, dotted line) and median true prevalence (TP, solid line) of detected pathogens in red deer at the *Oostvaardersplassen*. The figure shows AP and TP in percentages the years of 1999–2023. The shadow represents 95% uncertainty interval (UI) for TP. Each point represents a sampled year.

The number of pathogens detected in red deer decreased slightly over the study period compared to cattle. Antibodies reacting to the following viruses were found: BoHV-1 (both glycoproteins gB and gE) and BTV. Again, *F. hepatica* was the parasite detected and measured with serological diagnostic tests. Last, the bacteria observed were *S*. Dublin, *S*. Typhimurium and *MAP* (due to inconsistent testing, TP was not estimated).  Figure 4 and Table S4 show the variable AP and TP with corresponding 95% UIs and CIs. Furthermore, red deer were tested for chronic wasting disease (CWD), in accordance with international and national regulations, and no positive samples were identified.

Table 4 presents the findings on red deer parasites, with *Eimeria* spp. and Trichostrongylus/Strongylus eggs being the most frequently detected. *Moniezia expansa* was detected in only one case, while lungworm was not routinely investigated in red deer, except for one *D. viviparus* positive sample in 2018. *Eimeria* spp. was predominantly observed at ≤500 OPG (category ‘few'). Additionally, *Capillaria* sp. and *Trichuris* sp. were detected. Median EPG for Trichostrongylus/Strongylus eggs and *S. papillosus* per year was ≤200 (except 2003, median 300 EPG). Table S7 provides the number of samples, proportion of positive results per year and median EPG values for red deer.

#### 3.2.3. Konik Horses

**Figure 5:**
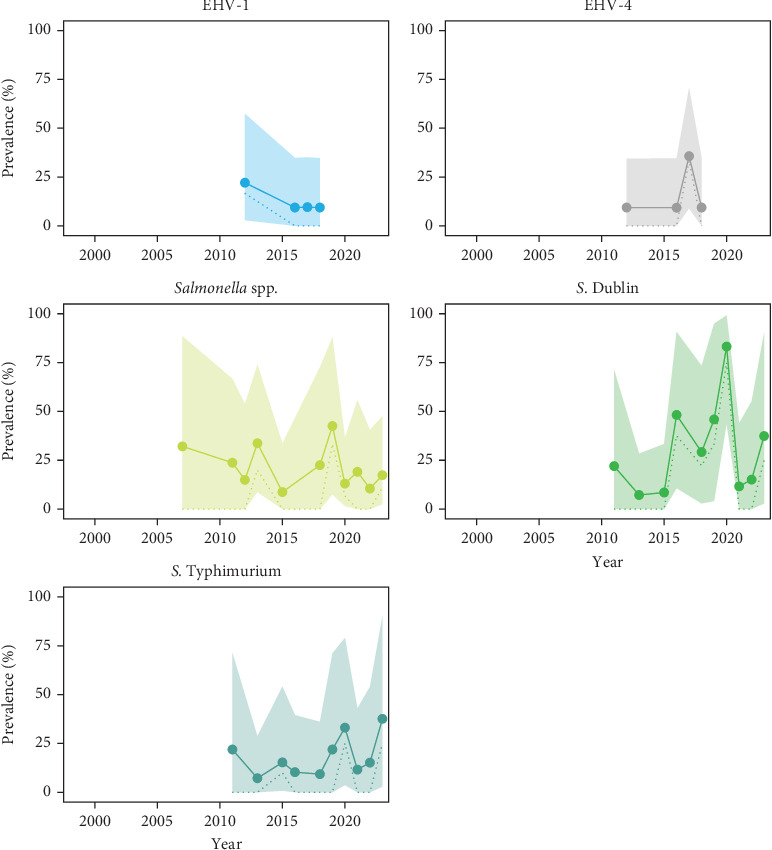
Apparent prevalence (AP, dotted line) and true prevalence (TP, solid line) of detected pathogens in Konik horses at the *Oostvaardersplassen*. The figure shows AP and TP in percentages between the years of 1999 and 2023. The shadow represents 95% uncertainty interval (UI) for TP. Each point represents a sampled year.

Horses were tested for horse-specific pathogens, except for salmonellas. The viruses detected were EHV-1 and EHV-4. The bacteria observed throughout the study period were *Salmonella* spp., *S*. Dublin and *S*. Typhimurium. Similarly to cattle, *Salmonella* spp. was observed, and four positive results were identified as *S*. Typhimurium and one as *Salmonella* group F-67. All these results are displayed in Figure 5, and in more detail in Table S5.

Regarding parasitic pathogens measured as EPG, Table 5 presents Konik horses parasites findings and the most frequently detected were Trichostrongylus/Strongylus eggs and *Parascaris* spp. *Eimeria* spp. was mostly observed at ≤500 OPG (category ‘few'). *Oxyuris* sp., *Paranoplocephala* sp. and *Anoplocephala* spp. were not observed. Table S8 provides the number of samples, proportion of positive results per year and median EPG values in horses. For Trichostrongylus/Strongylus eggs, median EPG was consistently >500.

### 3.3. Probability of Freedom From Infection

In cattle, the results for BLV and *L. i*. Hardjo indicated a high probability of freedom from infection (*p* free > 0.950) in the highest number of years, estimated in 18 out of the 26 years screened (69%). This was followed by *S*. Typhimurium (11 out of 23 years, 47%), *B. abortus* (nine out of 25 years, 36%), and bTB (six out of 25 years, 24%). FMDV was tested in only 2 years, with 1 year demonstrating a high probability of freedom (*p* free > 0.950) (Table S9). In the remaining years of testing, the number of samples submitted was insufficient to achieve adequate power to reject the *H*_0_ (detected prevalence ≥ DP); therefore, it was not possible to conclude whether the population was free from disease or distinguish it from one with a prevalence of 25%.

In red deer, although *C. burnetii* was tested in only 6 years, a high probability of freedom (*p* free > 0.950) at a 25% DP was demonstrated in half of those years (three out of six). This was followed by BVDV ag with 41% (seven out of 17 years), BVDV ab with 38% (seven out of 18 years), *B. abortus* with 33% (six out of 18 years), *L. i*. Hardjo with 20% (three out of 15 years), and *Salmonella* spp. with 6% (one out of 17 years). For both BLV and bTB, there was insufficient evidence in all tested years to conclude that the population was free from infection (Table S10).

Although in culture-based diagnostic testing bTB was not detected in cattle or red deer, some samples tested positive for other bacteria, specifically *MAP* or environmental non-pathogenic bacteria, such as *Mycobacterium fortuitum* and *Mycobacterium engbaekii*. In cattle, *MAP* was detected in two animals, *M. engbaekii* in three, *M. fortuitum* in 32 and unspecified *Mycobacterium* species in three. Red deer samples tested positive for *MAP* in two cases and for *M. fortuitum* in seven.

In horses, the viruses Equine infectious anaemia virus (EIAV), Equine influenza virus (EIV) and West Nile virus (WNV) were not detected. These pathogens were tested only in 3 years of the study period, and in all those years, there was insufficient evidence to conclude that the population was free from infection (Table S11).

For a more robust analysis of freedom from infection and a better estimate of prevalence, it is important to consider lower DPs that take into account pathogens present at lower levels, that is 5% and 10%. Assuming perfect diagnostic tests and applying Formula (1), the required sample size would increase to 28 samples per animal species for a target population of 300–500 animals at a 10% DP and to 54–56 samples at a 5% DP. However, when accounting for imperfect diagnostic test performance, the sample size estimates would need to be adjusted to between 27 and 208 samples per species at a 10% DP (Table 6).

## 4. Discussion

To assess the outcomes of the existing animal health monitoring system for free-living large herbivores in the OVP nature reserve in the Netherlands, monitoring efforts in cattle, horses and red deer were investigated. A retrospective longitudinal cross-sectional prevalence study identified various pathogens in all three species at different prevalence levels over the study period. Most detected pathogens are endemic to the Netherlands, while the absence of certain pathogens supports the country's free-status qualification.

BoHV-1 seropositivity at the OVP was higher when compared to a national survey [39], likely due to the non-interventionist management in the area, which does not include control measures used in livestock. The detected seropositivity primarily indicates long-lasting antibodies rather than recent infections, as BoHV-1 remains latent following infection and antibodies persist over time [40]. The high initial seroprevalence in cattle suggests infections may have occurred before their introduction to the OVP, as BoHV-1 is mostly transmitted through direct contact [40]. In red deer, BoHV-1 seroprevalence was higher than reported in red deer across Europe [41, 42], but it was consistent with the high cattle prevalence at the OVP. However, serological cross-reactivity with other ruminant herpesvirus, such as Cervid herpesvirus 1 (CvHV-1), known to circulate in European red deer [43, 44], could lead to false positive diagnoses. Additional testing, such as virus neutralisation test, could help further characterise the virus in red deer [41]. Moreover, glycoprotein E (gE) ELISA has been demonstrated to be able to differentiate between BoHV-1 and other ruminant herpesvirus [45]. This ELISA has been used at the OVP, with positive results supporting the presence of BoHV-1. Nonetheless, further research is needed to investigate the presence or absence of CvHV-1 at OVP. Last, red deer could potentially be reservoirs of BoHV-1. While seropositivity indicates exposure, an experimental study by Mollema et al. [46] suggested that the likelihood of transmission through direct contact between red deer and cattle is low, thus, red deer might not be contributing to BoHV-1 maintenance in cattle.

Both cattle and red deer at the OVP were tested for BTV, while only cattle were tested for SBV. A BTV serotype 8 outbreak occurred in the Netherlands from 2006 to 2008, with official free-status achieved in 2012, which was later revoked in 2023 due to a new outbreak of BTV serotype 3 [47]. At the OVP, BTV seroprevalence in cattle substantially increased in 2008, then gradually declined over subsequent years until 2022, not only likely reflecting long-lasting antibodies, but also indicating an apparent waning over time. In 2023, observed seropositivity increased, possibly indicating exposure to the new serotype. In red deer, seroprevalence was lower compared to cattle at the OVP and varied compared to deer species populations in other European countries [48, 49]. While no reports of BTV in red deer have been published in the Netherlands, roe deer (*Capreolus capreolus*) tested between 2009 and 2010 showed no evidence of antibodies [50]. Observations suggest red deer may be more susceptible to BTV infection than roe deer [48, 49], but their role in transmission may be limited following decline of BTV serotype 8 in livestock [51].

SBV was first detected near the Dutch-German border in 2011 and led to an outbreak that ended by 2013 [52]. At the OVP, SBV testing in cattle started in 2015, showing over 50% seroprevalence annually until 2023, comparable to observed seroprevalence during the outbreak in the Netherlands [53]. The high seroprevalence, particularly in earlier years, may reflect long-lasting immunity. However, seropositivity in cattle aged 1–2 years in 2020 and 2021 suggests possible new infections and virus recirculation at the OVP. This is consistent with the observation that since the emergence of SBV, the virus has shown a cyclical pattern of recirculation [54]. Although SBV testing was not carried out in red deer during the monitoring, it is recommended based on observations of this transmission pattern in red deer, among other species, in Europe [55, 56].

At the OVP, *MAP* was detected and confirmed to be circulating in both cattle and red deer, as positive cases were detected, with polymerase chain reaction (PCR) positive results in some instances. However, estimating TP was not feasible due to the inconsistent use of multiple diagnostic tests over and within the years, which would have led to less accurate prevalence estimates. The presence of *MAP* suggests that infections may have occurred prior to the introduction of animals to the OVP, as *MAP* is a chronic infection and diagnostic tests used may not have detected the pathogen due to poorer performance (Chapter 3.1.17. in [31]). While the first group of cattle introduced at the OVP was tested for *MAP*, the status of the later introduced groups and red deer was not recorded. Some deer were introduced from farms in the Netherlands, where *MAP* has been reported [57], and farmed cattle grazed in the area until 1995, suggesting possible introduction routes of *MAP* at the OVP. *MAP* has been reported in both cattle and red deer, in the Netherlands and abroad, with a possible interspecies transmission pattern from cattle to wild deer species and vice versa, especially when animals share pastures [58–61]. This transmission dynamic could explain the circulation of this bacterium at the OVP. However, further research is needed to assess to what extent these populations can be a risk for control in livestock.

*Fasciola hepatica* has a global distribution, including the Netherlands, and is found in semi-aquatic areas where its intermediary host is present [62]. At the OVP, the seroprevalence in cattle has been consistently high since 2007. However, earlier monitoring used diagnostic tests with lower performance, which may explain the lower seroprevalence in earlier years in the monitoring. *F. hepatica* was also detected in red deer, but at lower prevalence compared to cattle. As the OVP is a wetland area, the detection of *F. hepatica* in both animal species was expected. The higher prevalence in cattle compared to red deer is consistent with the results from other studies [63]. While it is debated whether deer species are an important reservoir responsible for the transmission of this parasite to livestock, it is suggested that this is unlikely as no correlations were found between roe deer and cattle [63].

In cattle, two additional pathogens were detected at lower prevalences: BVDV and *C. burnetii*. BVDV seroprevalence in Heck cattle at the OVP was low and comparable to nationally reported levels in livestock [39]. Like BoHV-1 and *MAP*, BVDV infections may have occurred before the animals were introduced to the OVP, potentially bringing persistently infected (PI) individuals into the population. Since PI animals are necessary for BVDV transmission, their presence is more likely in cattle populations without strict control programmes [64], such as the OVP. Additionally, ELISA for antigen detection is widely used for PI identification (Chapter 3.4.7. in [31]), and the detection of positive results using this test at the OVP confirms the presence of PI cattle. Although BVDV primarily affects cattle, interspecies transmission (e.g., deer species) can occur, which could potentially be the case at the OVP. However, no cases were detected, and other studies generally report lower prevalence in deer species than in livestock [42, 65–67]. Specifically, it has been demonstrated that while white-tailed deer (*Odocoileus virginianus*) can sustain infections and develop PI individuals, their role in cattle transmission appears limited [66]. Overall, most evidence suggests that BVDV risk in cattle is primarily driven by PI cattle, rather than spillback from deer, making it unlikely that deer species serve as significant reservoirs for cattle [42, 65, 66].

*Coxiella burnetii* was detected in cattle at lower prevalence when compared with reported seroprevalence in dairy cattle in the Netherlands [68] and aligns with findings in other European countries, where lower seroprevalence was observed in wild ruminants compared with domestic ruminants [69].

With respect to *Salmonella* species, in this study animals were tested primarily for *S*. Dublin and *S*. Typhimurium, since these two serovars are common in the Netherlands and routinely monitored [39]. Both serovars were detected in cattle, red deer and horses. Intermittent detection of *Salmonella* spp. at the OVP may be attributed to the presence of asymptomatic carriers that shed bacteria intermittently, meaning a single negative test does not necessarily confirm the absence of infection. This is particularly relevant when using culture-based methods with low Se, as low bacterial levels in a sample may go undetected (Chapter 3.10.3. in [31]). Serological tests for specific *Salmonella* serovars generally offer higher diagnostic performance and can indicate exposure. However, these tests have limited Sp, which increases the likelihood of false positives, particularly in low-prevalence settings (Chapter 3.10.3. in [31]), which characterises the Netherlands [70].

EHV-1 and EHV-4 were detected in horses at the OVP. The detection of these equine viruses was not surprising as these viruses are common equine pathogens, with high seroprevalence reported in the country [71] and documented occurrences in other wild equid species [72]. At the OVP, PCR was used as the diagnostic method for Konik horses. While PCR is highly sensitive and specific for confirming clinical cases, VNT and ELISA are recommended for surveillance purposes (Chapter 3.6.8. in [31]). Since the monitoring system aims to assess pathogen presence and prevalence rather than diagnose clinical cases, adopting serological tests would provide a more accurate estimation of infection prevalence in the nature reserve.

The presence of parasites at the OVP, particularly strongyles, was expected due to the ubiquitous nature of these pathogens, continuous year-round grazing, and the absence of antiparasitic control measures. Additionally, given that many of these parasites are known to infect both domestic and wild ungulates [73], the presence of the parasites from the same class (i.e., Nematoda) indicates potential for parasite persistence in the environment in the nature reserve. Further investigation with larger sample sizes and increased testing frequency would provide a clearer picture of parasite seasonal dynamics in the area and potential health implications for OVP populations.

Among the pathogens not detected during the study period, BLV showed high probability of freedom from infection in cattle, but evidence was insufficient for red deer. Confidence in freedom was lower for *B. abortus* and bTB. However, all three pathogens have had national free-status since 1999, with no reported infections in livestock since then [74]. Although the statistical power is insufficient to definitively confirm freedom from infection, it is reasonable to assume that OVP populations are also free of these pathogens.

For *L. i*. Hardjo, confidence in freedom was higher in cattle than in red deer. Although, *Leptospira* spp. has been reported in deer species [75, 76], its absence in both species at the OVP aligns with very low national prevalence [39]. However, one limitation of the test used is that it cannot detect other pathogenic *Leptospira* species. FMDV was not detected in cattle in the 2 years of testing, as expected, given the Netherlands has maintained free-status since regaining it after the 2001 epidemic [77]. Similarly, the risk of BSE is considered negligible, with no reported cases in the country since 2010 [74].

In red deer, BVDV was determined to be absent in 40% of the years that the pathogen was tested, consistent with low prevalence in other European countries [42, 65, 67], suggesting that red deer at the OVP do not seem to be reservoirs for BVDV or a source for cattle infection due to the reasons previously mentioned. The absence of *C. burnetii* contrasts with findings from other studies, as *C. burnetti* was retrospectively detected in roe deer during the Dutch epidemic [78] and at lower concentrations in deer species in other European countries [79, 80]. High confidence of freedom was demonstrated in only half of the tested years, meaning absolute certainty of freedom cannot be determined, especially given the pathogen's presence in cattle. As for CWD, no cases were detected, which was expected, as the disease has so far been limited to Nordic countries [81, 82].

Regarding horses, EIAV, EIV and WNV were not detected in limited years of testing. However, due to insufficient evidence, freedom from infection could not be concluded for any of these viruses. Recent surveillance efforts and reported cases for these viruses suggest continued monitoring at the OVP is essential [83, 84].

This study provides valuable insights into pathogen prevalence among cattle, red deer and horses. However, some methodological considerations should be acknowledged. Sample sizes varied between years, ranging from three to approximately 30, which may have contributed to variations in prevalence estimates in some pathogens (e.g., Salmonellas). While using a Bayesian framework helped account for limited sample sizes and imperfect diagnostic tests, in some cases, wider credible intervals indicate a degree of uncertainty in the estimates. In years with fewer samples, confidence in determining freedom from infection was also lower, highlighting the importance of consistent and sufficiently large sample sizes for reliable long-term trends. Additionally, shifts in demographic composition could have influenced prevalence estimates, though further analysis was limited due to a high number of unknown age in the data. Despite these considerations, this study demonstrates the feasibility of pathogen monitoring in free-living herbivores in a nature reserve and provides a valuable start for future research. Expanding and standardising sampling efforts will further strengthen prevalence estimates, improve confidence in estimation of freedom from infection, and improve the understanding of infection dynamics in these populations.

Furthermore, while diagnostic test performance was considered in TP estimation in the current study, most Se and Sp values were based on tests used and validated in livestock, which may not accurately apply to wild species such as red deer. Test accuracy in wildlife remains uncertain due to species diversity, pathogen variability, and limited validation data. Therefore, estimates in low-prevalence settings should be cautiously interpreted [85, 86]. To address this, Bayesian latent class models are increasingly used to estimate diagnostic test performance in wild species [86].

The ongoing active monitoring system at the OVP is highly valuable and informative, but some points for improvement have been identified. Throughout the study period, sampling strategies have included a combination of random, purposive, and convenience sampling, which can introduce selection bias and limit the generalizability of results [2]. In some cases, weaker-looking animals may have been sampled more frequently due to ease of capture, potentially increasing the probability of detecting disease. While this aligns with the monitoring system's goal of pathogen detection, it may not provide a fully representative assessment of the broader population. Additionally, sample collection has not been evenly distributed across and within the years, with a higher concentration of samples obtained in late winter and early spring, which can limit the analysis of seasonal dynamics. To address these limitations, a harmonised testing protocol with regular assessments of relevant pathogens is essential. This protocol should ensure consistent testing across species and samples, using standardised diagnostic methods each year while allowing for the inclusion of new pathogens as needed. Increasing sample sizes, particularly for Konik horses and red deer, is crucial to improve the accuracy and representativeness of prevalence estimates, ensuring all three animal populations receive equal consideration in monitoring efforts.

Current monitoring efforts focus on detecting pathogen presence or absence based on a 25% DP, which could be considered as a relatively moderate to high given the impact and importance of the pathogens assessed. However, this threshold has not been sufficient to confidently conclude pathogen absence. Reducing the DP and increasing the sample size could improve confidence in prevalence estimates or confirm freedom from infection, resulting in a more reliable system and generating more representative data. Nevertheless, larger sample sizes pose operational challenges and might reduce cost-effectiveness in OVP management.

Given pathogen and population dynamics, prioritisation of pathogens tested for all three animal species at the OVP should be reviewed. Although this process has not yet been carried out, such evaluations are essential, despite the challenges posed by the free-living nature of these animals. In Europe, efforts have been made to establish a list of prioritised pathogens for wildlife, many of which can infect ruminants, including red deer [10, 87]. The current monitoring at the OVP includes eight of these pathogens (*S. enterica*, *C. burnetii*, FMDV, bTB, BTV, *B. abortus*, *MAP* and *Leptospira* sp.), which have been tested either consistently or at least once during the study period. However, other noted relevant pathogens, such as hepatitis E virus, tick-borne encephalitis virus, *Babesia sp*., and *Anaplasma phagocytophilum*, are not yet included in the monitoring system. Additionally, some pathogens specific to horses, such as WNV, were tested for a short period but have not been monitored in recent years. Considering the emergence and re-emergence of various pathogens over the past decade, a reassessment of testing priorities should not only focus on pathogens that threaten livestock, but also consider those impacting wildlife populations and/or have zoonotic potential.

Significant outbreaks, such as BTV in the Netherlands [47], EHDV in France [88] and H5N1 in the United States [89], highlight the importance to prioritise and introduce new pathogens in the monitoring system, based on emerging reports within national and regional contexts. Updating the pathogen panel for testing is vital not only for safeguarding animal health and welfare among free-living herbivores and their domestic counterparts but also for public health consequences. These animals may serve as sentinel species for various pathogens, emphasising the need for an adaptive surveillance strategy responsive to evolving epidemiological settings.

## 5. Conclusions

This study represents the first assessment of pathogen prevalence among free-living large herbivores in a nature reserve in the Netherlands. Findings show that both cattle, horses and red deer contribute to maintaining various endemic and zoonotic pathogens in this ecosystem. This underscores the need for further research in other nature reserves to assess how wild populations affect national disease control programmes in domestic livestock and to identify feasible intervention strategies.

The existing monitoring system at the OVP already captures valuable insights of presence of pathogens in nature reserves, especially where multiple animal species coexist. Enhancing this system through increased sample sizes and prioritising specific pathogens for each species could improve its effectiveness and overall value.

From a One Health perspective, the current findings reinforce the importance of including free-living and wild animals in national surveillance efforts and establishing similar monitoring systems in other nature reserves. This approach will not only strengthen our understanding of free-living and wild animal health, but also help mitigate potential risks to public health and livestock.

## Figures and Tables

**Table 1:** Overall list of pathogens (viruses, prions, bacteria and parasites) included in monitoring programme at the *Oostvaardersplassen*.

Type of pathogen	Pathogens tested	Animal species
Viruses	Bovine herpesvirus 1 (BoHV-1 gB and gE) Bovine leukaemia virus (BLV) Bovine viral diarrhoea virus antibodies and antigen (BVDV ab and ag) Bluetongue virus (BTV)	Cattle Red deer
	Foot and mouth disease virus (FMDV) Schmallenberg virus (SBV)	Cattle
	Equine herpesvirus 1 (EHV-1) Equine herpesvirus 4 (EHV-4) Equine infectious anaemia virus (EIAV) Equine influenza virus (EIV) West Nile virus (WNV)	Horses

Prions	Bovine spongiform encephalopathy (BSE)	Cattle
	Chronic wasting disease (CWD)	Red deer

Bacteria	*Brucella abortus* *Coxiella burnetii* *Leptospira interrogans* serovar Hardjo (*L. i*. Hardjo) *Mycobacterium paratuberculosis* subsp. a*vium* (*MAP*) *Mycobacterium bovis* (bTB)	Cattle Red deer
	*Salmonella* spp. *Salmonella enterica* serovar Dublin (*S*. Dublin) *Salmonella enterica* serovar Typhimurium (*S*. Typhimurium)	Cattle Red deer Horses

Parasites	*Fasciola hepatica* Lungworm *Moniezia expansa* *Nematodirus battus* *Nematodirus* spp. *Strongyloides papillosus*	Cattle Red deer
	Ectoparasites *Eimeria* spp. Other endoparasites Trichostrongylus/Strongylus eggs	Cattle Red deer Horses
	*Anoplocephala* spp. *Oxyuris* sp. *Paranoplocephala* sp. *Parascaris* sp. *Strongyloides westeri*	Horses

**Table 2:** Sex and age group proportions for Heck cattle, red deer and Konik horses with the inclusion of all samples for the total study period.

Categories	Cattle	Red deer	Horses
Total, *n*	554	190	240
Sex, *n* (*p*)			
Female	237 (0.43)	80 (0.42)	66 (0.28)
Male	81 (0.14)	47 (0.25)	62 (0.26)
Unknown	236 (0.43)	63 (0.33)	112 (0.46)
Age group, *n* (*p*)			
<1 year	30 (0.05)	30 (0.16)	22 (0.09)
1–2 years	71 (0.13)	30 (0.16)	26 (0.11)
2–5 years	84 (0.15)	13 (0.07)	32 (0.13)
5–10 years	85 (0.15)	7 (0.04)	31 (0.13)
2–10 years^a^	—	37 (0.19)	—
>10 years	47 (0.09)	8 (0.04)	20 (0.08)
Unknown	237 (0.43)	65 (0.35)	109 (0.45)

*Note: n* is number of sampled animals and *p* the proportion of sampled animals.

^a^Age group for female red deer as it was not possible to further classify due to lack of antlers.

**Table 3:** Overview of parasites tested in Heck cattle during the study period.

Year/pathogen	1998	1999	2000	2001	2002	2003	2004	2005	2006	2007	2008	2009	2010	2011	2012	2013	2015	2017	2018	2019	2020	2021	2022	2023
Ectoparasites	**P**				**P**																		–	–
*Eimeria* spp.		○	○	○	○	●	○	●	○	○	○	○	●	○	○	–	–	–	–	–	○	○	○	–
*Moniezia. expansa*		–	–	–	–	–	–	–	–	–	–	–	**P**	–	–	–	–	–	–	–	**P**	–	–	**P**
Lungworm^a^	–	–	**P**			**P**																		
*Nematodirus* spp.		○	–	–	–	○	–	○	○	–	–	–	–	–	–	○	–	–	–	–	–	–	–	–
*N. battus*		–	–	–	–	–	–	–	–	–	–	–	–	–	–	–	–	–	–	–	–	–	–	–
Trichostrongylus/Strongylus eggs		●	○	●	○	●	●	●	●	●	●	●	●	○	○	○	○	–	–	–	○	–	○	○
*S. papillosus*		○	○	○	○	–	○	–	○	–	○	○	○	○	○	–	○	–	–	–	–	○	○	–
Other endoparasites^a^		–	–	**P**	–	–	–	–	–	**P**	**P**	–	–	–	–	–	–	–	–	–	–	–	–	–

*Note:* Empty cells indicate not tested. “–” tested negative; “ **P** ” tested positive (no EPG count); “●” tested positive, more than one animal with EPG > 200 EPG (Eimeria > 500 OPG); “○” tested positive, EPG < 200 EPG (Eimeria < 500 OPG).

^a^Lungworm was identified as *Dictyocaulus viviparus*; other endoparasites detected were all *Capillaria* sp.

**Table 4:** Overview of parasites tested in red deer during the study period.

Year/pathogen	1998	1999	2000	2001	2002	2003	2004	2005	2006	2007	2008	2009	2010	2011	2012	2013	2015	2017	2018	2019	2020	2021	2022	2023
Ectoparasites																							–	–
*Eimeria* spp.			–	○	–	–		○		○				○	○	○	●	–	○	–	○	–	○	○
*Moniezia. expansa*			–	–	–	–		–		–				–	–	–	–	–	–	**P**	–	–	–	–
*Nematodirus* spp.			–	–	–	–		–		–				–	–	–	–	–	–	–	–	–	–	–
*N. battus*			–	–	–	–		–		–				–	–	–	–	–	–	–	–	–	–	–
Trichostrongylus/Strongylus eggs			○	○	–	○		○		●				○	○	●	○	○	○	–	○	○	○	○
*S. papillosus*			–	–	–	–		–		–				–	–	○	–	–	–	○	○	–	○	○
Other endoparasites^a^			–	–	–	–		–		–				–	–	**P**	–	–	–	–	**P**	–	**P**	–

*Note:* Empty cells indicate not tested. “–” tested negative; “ **P** ” tested positive (no EPG count); “●” tested positive, more than one animal with EPG > 200 EPG (Eimeria > 500 OPG); “○” tested positive, EPG < 200 EPG (Eimeria < 500 OPG).

^a^Other endoparasites were identified as *Capillaria* sp. and *Trichuris* sp.

**Table 5:** Overview of parasites tested in Konik horses during the study period.

Year/pathogen	1998	1999	2000	2001	2002	2003	2004	2005	2006	2007	2008	2009	2010	2011	2012	2013	2015	2017	2018	2019	2020	2021	2022	2023
Ectoparasites																							–	–
*Eimeria* spp.														–	○	–	–			–	○			–
*Anoplocephala* spp.		–	–	–	–	–	–	–		–				–	–	–	–		–	–	–	–	–	–
*Paranoplocephala* sp.		–	–	–	–	–	–	–		–				–	–	–	–		–	–	–	–	–	–
*Parascaris* sp.		–	●	–	–	○	●	○		●				○	–	○	○		–	–	●	–	–	–
*Oxyuris* sp.		–	–	–	–	–	–	–		–				–	–	–	–		–	–	–	–	–	–
Trichostrongylus/Strongylus eggs		–	●	●	●	●	●	●		●				○	○	○	○		●	●	●	●	●	●
*S. westeri*		–	–	–	–	–	○	–		–				–	○	○	○		–	–	–	–	–	–
Other endoparasites		–	–	–	–	–	–	–		–				–	–	–	–		–	–	–	–	–	–

*Note:* Empty cells indicate not tested. “–” tested negative; “●” tested positive, more than one animal with EPG > 200 EPG (Eimeria > 500 OPG); “○” tested positive, EPG < 200 EPG (Eimeria < 500 OPG).

**Table 6:** Required sample size (*n*) based on imperfect diagnostic tests to classify freedom from infection in Heck cattle, red deer and Konik horse populations, based on the target population size for each species at the *Oostvaardersplassen* at 10% design prevalence (DP).

Pathogen (diagnostic test)	Heck cattle (*N* = 300)	Red deer (*N* = 500)	Konik horses (*N* = 300)
*B. abortus* (RBT)	44	45	—
BLV (ELISA)	42	43	—
BoHV-1 (ELISA)	43	44	—
bTB (Culture)	36	36	—
BTV (ELISA)	27	28	—
BVDV ag (ELISA)	42	43	—
*C. burnetii* (CFT)	30	30	—
*Fasciola hepatica* (ELISA)	51	52	—
*L. i*. Hardjo (ELISA)	43	44	—
*MAP* (ELISA)	56	57	—
*Salmonella* spp. (MALDI−TOF (with enrichment))	40	41	40
*S*. Dublin (TSAT)	203	206	203
*S*. Typhimurium (TSAT)	203	206	203
SBV (ELISA)	81	—	—
EHV-1 and 4 (PCR)	—	—	53
EIAV (ELISA)	—	—	28
EIV (HI)	—	—	29
WNV (ELISA)	—	—	29

## Data Availability

The data that were analysed during the current study are available from *Staatsbosbeheer*, but restrictions apply to the availability of these data and thus are not publicly available. However, data are available upon reasonable request and with permission of *Staatsbosbeheer* (contact person: Perry Cornelissen p.cornelissen@staatsbosbeheer.nl).

## Supporting Information

Supporting Information Table S1: Categories of diseases for the OVP animal health monitoring system in 1997. Table S2: Sensitivity and specificity values per diagnostic test and pathogen. Table S3: Apparent and true prevalence for Heck cattle. Table S4: Apparent and true prevalence for red deer. Table S5: Apparent and true prevalence for Konik horses. Table S6: Overview of Heck cattle parasite results. Table S7: Overview of red deer parasite results. Table S8: Overview of Konik horses parasite results. Table S9: Results of probability of freedom from infection analysis in Heck cattle. Table S10: Results of probability of freedom from infection analysis in red deer. Table S11: Results of probability of freedom from infection analysis in Konik horses.
