# Supplementary material for: Monitoring Pathogens in Free-Living Large Herbivores in a Nature Reserve in the Netherlands
Source: Transbound Emerg Dis. 2025 Aug 8;2025:6948049. doi: 10.1155/tbed/6948049 (PMC12356683; doi:10.1155/tbed/6948049)
Supplement: Supporting Information — Table S1: Categories of diseases for the OVP animal health monitoring system in 1997. Table S2: Sensitivity and specificity values per diagnostic test and pathogen. Table S3: Apparent and true prevalence for Heck cattle. Table S4: Apparent and true prevalence for red deer. Table S5: Apparent and true prevalence for Konik horses. Table S6: Overview of Heck cattle parasite results. Table S7: Overview of red deer parasite results. Table S8: Overview of Konik horses parasite results. Table S9: Results of probability of freedom from infection analysis in Heck cattle. Table S10: Results of probability of freedom from infection analysis in red deer. Table S11: Results of probability of freedom from infection analysis in Konik horses. [file 6948049.f1.docx]

# Supplementary materials: Monitoring pathogens in free-living large herbivores in a nature reserve in the Netherlands

Inês Marcelino^1^, Jasmin Keizer^1^, Gustavo Monti^1^, Perry Cornelissen^2,3^, Inge Santman-Berends^4^, Jasper het Lam^5^, Wim H.M. Van der Poel^1,6^

^1^ Infectious Disease Epidemiology (IDE), Wageningen University and Research (WUR), Wageningen, the Netherlands

^2^ Department Nature & Society, Staatsbosbeheer, Lelystad, the Netherlands

^3^ Institute for Biodiversity and Ecosystem Dynamics, University of Amsterdam (UvA), Amsterdam, the Netherlands

^4^ Department of Research and Development, Royal GD, Deventer, the Netherlands

^5^ Ruminant Health Department, Royal GD, Deventer, the Netherlands

^6^ Wageningen Bioveterinary Research (WBVR), Lelystad, the Netherlands

Table S1– Categories and respective descriptions for classification of diseases included in the animal health monitoring programme at the OVP in 1997 (Hessels, 1997).

| Category | Description | Examples |
| --- | --- | --- |
| I | Notifiable cattle diseases for which a monitoring system, based on clinical suspicion, followed by laboratory diagnostics, is used in the Netherlands to monitor the Dutch status regarding these diseases. | FMDV  Rabies  Anthrax  BSE |
| II | Cattle diseases that are not endemic in the Netherlands and for which there is a monitoring system in the Netherlands to guarantee freedom from these diseases. Domestic outbreaks of these diseases are mainly caused by the import of infected animals from abroad. | *B. abortus*  bTB  BLV |
| IIIa | Cattle diseases endemic to the Dutch cattle population, for which the Netherlands will implement control programmes in the near future to achieve the “disease-free” status. | BoHV-1  *L.* Hardjo |
| IIIb | Cattle diseases endemic to the Dutch cattle population, for which the Netherlands may draw up control programmes in the future. | MAP  BVDV  *S.* Dublin  *S.* Typhimurium |
| IV | Cattle diseases endemic in the Dutch cattle population, which can form a parameter in the context of animal health and welfare. | Endoparasites  Ectoparasites |

Table S2 – Sensitivity (Se) and specificity (Sp) values per diagnostic test per pathogen used in all three animal species (Heck cattle, red deer, Konik horses).

| Pathogen | Method | Se | Sp | Reference |
| --- | --- | --- | --- | --- |
| *B. abortus* | CFT | 96.0 | 99.8 | (Greiner et al., 2009) |
|  | ELISA | 100 | 99.6 | (Greiner et al., 2009) |
|  | MAT | 81.7 | 98.7 | (Greiner et al., 2009) |
|  | RBT | 98.1 | 99.8 | (Greiner et al., 2009) |
|  | Culture | 21.6* | 100 | (A. Michel, personal communication, 2024) |
| BLV | AGIDT | 79.7 | 99.0 | (Trono et al., 2001) |
|  | ELISA | 99.6 | 99.6 | GD Animal Health |
| BoHV-1 gB | ELISA | 99.0 | 99.7 | (de Wit et al., 1998; Kramps et al., 1994) |
| BoHV-1 gE | ELISA | 99.0 | 100 | (de Wit et al., 1998) |
| bTB | Culture | 78.1 | 100 | WBVR & (Courcoul et al., 2014) |
|  | Ziehl Neelsen | 33.9 | 100 | (Varello et al., 2008) |
| BTV | ELISA | 100 | 99.9 | GD Animal Health |
| BVDV ab | ELISA | 98.0 | 99.0 | GD Animal Health |
| BVDV ag | ELISA | 99.0 | 99.5 | GD Animal Health |
| *C. burnetii* | CFT | 92.5* | 100 | WBVR |
|  | ELISA | 90.0 | 98.0 | GD Animal Health |
| EHV-1 and 4 | PCR | 100 | 0.99 | (Diallo et al., 2006) |
| EIAV | AGIDT | 100 | 99.7 | WBVR |
|  | ELISA | 98.8* | 100 | (Nardini et al., 2017) |
| EIV | HI | 95.0 | 100 | (Vila Roza et al., 2000) |
|  | ELISA | 98.5* | 95.4* | (Kittelberger et al., 2011) |
| *F. hepatica* | ELISA | 99.4 | 98.5 | GD Animal Health |
|  | IHA | 57.0 | 85.0 | GD Animal Health |
| FMDV | ELISA | 98.3 | 99.2 | WBVR |
| *L.* Hardjo | ELISA | 97.6 | 99.6 | GD Animal Health |
|  | MAT | 67.0 | 97.0 | (Bajani et al., 2003; World Organisation for Animal Health (WOAH), 2021a) |
| *MAP* | CFT | 96.8* | 91.5* | WBVR |
|  | Culture | 26.0* | 100 | (World Organisation for Animal Health (WOAH), 2021b) |
|  | ELISA | 77.0 | 99.8 | GD Animal Health & (Weber et al., 2009) |
|  | PCR | 95.0 | 95.0 | GD Animal Health |
|  | Ziehl Neelsen | 49.3 | 83.3 | (Weber et al., 2009) |
| *Salmonella* spp. | Culture/MALDI-TOF (with enrichment) | 70.0 | 100 | GD Animal Health |
|  | Culture/MALDI-TOF (without enrichment) | 20.0 | 100 | GD Animal Health |
|  | ELISA | 94.0 | 99.3 | GD Animal Health |
| *S.* Dublin | TSAT | 73.8 | 93.2 | (Sánchez-Miguel et al., 2018) |
| *S.* Typhimurium | TSAT | 73.8 | 93.2 | (Sánchez-Miguel et al., 2018) |
| SBV | ELISA | 98.8 | 96.0 | GD Animal Health |
| WNV | ELISA | 96.5 | 100 | (Sotelo et al., 2011) |

**Key –** *denotes an average estimate; **Pathogens:** *B. abortus* = *Brucella abortus;* BLV = Bovine leukaemia virus; BoHV-1 = Bovine Herpesvirus 1; BTV = Bluetongue virus; BVDV ab = Bovine viral diarrhoea virus antibodies; BVDV ag = Bovine viral diarrhoea virus antigen; *C. burnetii* = *Coxiella burnetii* (Q fever); EHV1 and 4 = Equine Herpesvirus 1 and 4; EIAV = Equine Infectious Anaemia Virus; EIV = Equine influenza virus; *F. hepatica* = *Fasciola hepatica*; FMDV = Foot and mouth disease virus; *L.* Hardjo = *Leptospira interrogans* serovar Hardjo; *MAP* = *Mycobacterium paratuberculosis* subsp. *avium*; bTB = *Mycobacterium bovis* (Bovine Tuberculosis); *S.* Dublin = *Salmonella enterica* serovar Dublin; *S.* Typhimurium = *Salmonella enterica* serovar Typhimurium; SBV = Schmallenberg virus; WNV = West Nile Virus; **Methods:** AGIDT = agar gel immunodiffusion test; CFT = complement fixation test; ELISA = enzyme-linked immunosorbent assay; HI = haemagglutination inhibition test; IHA = indirect haemagglutination test; MALDI-TOF = matrix-assisted laser desorption – ionisation-time of flight mass spectrometry; MAT = micro agglutination test; PCR = polymerase chain reaction; RBT = rose bengal test; TSAT = tube serum agglutination test.

**References:**

Bajani, M. D., Ashford, D. A., Bragg, S. L., Woods, C. W., Aye, T., Spiegel, R. A., Plikaytis, B. D., Perkins, B. A., Phelan, M., Levett, P. N., & Weyant, R. S. (2003). Evaluation of four commercially available rapid serologic tests for diagnosis of leptospirosis. *Journal of Clinical Microbiology*, *41*(2), 803–809. https://doi.org/10.1128/JCM.41.2.803-809.2003

Courcoul, A., Moyen, J.-L., Brugère, L., Faye, S., Hénault, S., Gares, H., & Boschiroli, M.-L. (2014). Estimation of sensitivity and specificity of bacteriology, histopathology and PCR for the confirmatory diagnosis of bovine tuberculosis using latent class analysis. *PloS One*, *9*(3), e90334. https://doi.org/10.1371/journal.pone.0090334

de Wit, J. J., Hage, J. J., Brinkhof, J., & Westenbrink, F. (1998). A comparative study of serological tests for use in the bovine Herpesvirus 1 eradication programme in The Netherlands. *Veterinary Microbiology*, *61*(3), 153–163. https://doi.org/10.1016/S0378-1135(98)00166-7

Diallo, I. S., Hewitson, G., Wright, L., Rodwell, B. J., & Corney, B. G. (2006). Detection of equine herpesvirus type 1 using a real-time polymerase chain reaction. *Journal of Virological Methods*, *131*(1), 92–98. https://doi.org/10.1016/j.jviromet.2005.07.010

Greiner, M., Verloo, D., & de Massis, F. (2009). Meta-analytical equivalence studies on diagnostic tests for bovine brucellosis allowing assessment of a test against a group of comparative tests. *Preventive Veterinary Medicine*, *92*(4), 373–381. https://doi.org/10.1016/j.prevetmed.2009.07.014

Hessels, A. L. D. (1997). *Protocollaire aanpak ter bepaling, bewaking en beheersing van mogelijke besmettelijke dierziekten bij de Heckrunderen in de Oostvaardersplassen.* Faculteit Diergeneeskunde Universiteit van Utrecht.

Kittelberger, R., McFadden, A. M. J., Hannah, M. J., Jenner, J., Bueno, R., Wait, J., Kirkland, P. D., Delbridge, G., Heine, H. G., Selleck, P. W., Pearce, T. W., Pigott, C. J., & O’Keefe, J. S. (2011). Comparative evaluation of four competitive/blocking ELISAs for the detection of influenza A antibodies in horses. *Veterinary Microbiology*, *148*(2), 377–383. https://doi.org/10.1016/j.vetmic.2010.08.014

Kramps, J. A., Magdalena, J., Quak, J., Weerdmeester, K., Kaashoek, M. J., Maris-Veldhuis, M. A., Rijsewijk, F. A., Keil, G., & van Oirschot, J. T. (1994). A simple, specific, and highly sensitive blocking enzyme-linked immunosorbent assay for detection of antibodies to bovine herpesvirus 1. *Journal of Clinical Microbiology*, *32*(9), 2175–2181. https://doi.org/10.1128/jcm.32.9.2175-2181.1994

Nardini, R., Autorino, G. L., Issel, C. J., Cook, R. F., Ricci, I., Frontoso, R., Rosone, F., & Scicluna, M. T. (2017). Evaluation of six serological ELISA kits available in Italy as screening tests for equine infectious anaemia surveillance. *BMC Veterinary Research*, *13*(1), 105. https://doi.org/10.1186/s12917-017-1007-6

Sánchez-Miguel, C., Crilly, J., Grant, J., & Mee, J. F. (2018). Sensitivity, specificity and predictive probability values of serum agglutination test titres for the diagnosis of Salmonella Dublin culture-positive bovine abortion and stillbirth. *Transboundary and Emerging Diseases*, *65*(3), 676–686. https://doi.org/10.1111/tbed.12784

Sotelo, E., Llorente, F., Rebollo, B., Camuñas, A., Venteo, A., Gallardo, C., Lubisi, A., Rodríguez, M. J., Sanz, A. J., Figuerola, J., & Jiménez-Clavero, M. Á. (2011). Development and evaluation of a new epitope-blocking ELISA for universal detection of antibodies to West Nile virus. *Journal of Virological Methods*, *174*(1), 35–41. https://doi.org/10.1016/j.jviromet.2011.03.015

Trono, K. G., Pérez-Filgueira, D. M., Duffy, S., Borca, M. V., & Carrillo, C. (2001). Seroprevalence of bovine leukemia virus in dairy cattle in Argentina: Comparison of sensitivity and specificity of different detection methods. *Veterinary Microbiology*, *83*(3), 235–248. https://doi.org/10.1016/S0378-1135(01)00420-5

Varello, K., Pezzolato, M., Mascarino, D., Ingravalle, F., Caramelli, M., & Bozzetta, E. (2008). Comparison of histologic techniques for the diagnosis of bovine tuberculosis in the framework of eradication programs. *Journal of Veterinary Diagnostic Investigation: Official Publication of the American Association of Veterinary Laboratory Diagnosticians, Inc*, *20*(2), 164–169. https://doi.org/10.1177/104063870802000204

Vila Roza, M. V., Galosi, C. M., Oliva, G. A., Echeverría, M. G., Pecoraro, M. R., Corva, S., & Etcheverrigaray, M. E. (2000). ELISA indirecto para el diagnóstico rápido de Influenza Equina [Indirect ELISA for the rapid diagnosis of Equine Influenza]. *Revista Argentina De Microbiologia*, *32*(1), 39–43.

Weber, M. F., Verhoeff, J., van Schaik, G., & van Maanen, C. (2009). Evaluation of Ziehl-Neelsen stained faecal smear and ELISA as tools for surveillance of clinical paratuberculosis in cattle in the Netherlands. *Preventive Veterinary Medicine*, *92*(3), 256–266. https://doi.org/10.1016/j.prevetmed.2009.08.017

World Organisation for Animal Health (WOAH). (2021a). *Manual of Diagnostic Tests and Vaccines for Terrestrial Animals, Chapter 3.1.12. Leptospirosis*. WOAH - World Organisation for Animal Health. https://www.woah.org/en/what-we-do/standards/codes-and-manuals/terrestrial-manual-online-access/

World Organisation for Animal Health (WOAH). (2021b). *Manual of Diagnostic Tests and Vaccines for Terrestrial Animals, Chapter 3.1.16. Paratuberculosis (Johne’s Disease)*. WOAH - World Organisation for Animal Health. https://www.woah.org/en/what-we-do/standards/codes-and-manuals/terrestrial-manual-online-access/

## Prevalence results

Table S3 – Apparent prevalence (AP) with Wilson’s 95% confidence interval (CI) and median true prevalence (TP) with 95% uncertainty interval (UI) per pathogen per year for Heck cattle. *N* indicates total population, *n* sample population and *x* number of positive samples.

| **Pathogen** | **Year** | **N** | **n** | **x** | **AP (CI) %** | **TP (UI) %** |
| --- | --- | --- | --- | --- | --- | --- |
| **BoHV-1 gB** | 1998 | 434 | 15 | 13 | 86.7 (62.1 – 96.3) | 84.5 (62.1 – 96.9) |
|  | 1999 | 442 | 20 | 14 | 70.0 (48.1 – 85.5) | 69.4 (48.2 – 86.3) |
|  | 2000 | 500 | 22 | 20 | 90.9 (72.2 – 97.5) | 89.4 (72.5 – 98.2) |
|  | 2001 | 486 | 17 | 15 | 88.2 (65.7 – 96.7) | 86.3 (65.9 – 97.4) |
|  | 2002 | 539 | 13 | 12 | 92.3 (66.7 – 99.6) | 89.1 (66.6 – 98.9) |
|  | 2003 | 485 | 19 | 12 | 63.2 (41.0 – 80.9) | 62.8 (41.0 – 81.5) |
|  | 2004 | 573 | 6 | 6 | 100.0 (61.0 – 100.0) | 90.6 (65.0 – 100.0) |
|  | 2005 | 442 | 20 | 12 | 60.0 (38.7 – 78.1) | 59.7 (38.5 – 78.8) |
|  | 2006 | 379 | 13 | 10 | 76.9 (49.7 – 91.8) | 75.1 (49.5 – 92.6) |
|  | 2007 | 379 | 20 | 9 | 45.0 (25.8 – 65.8) | 45.5 (25.6 – 66.7) |
|  | 2008 | 404 | 15 | 8 | 53.3 (30.1 – 75.2) | 53.4 (30.1 – 76.2) |
|  | 2009 | 311 | 17 | 12 | 70.6 (46.9 – 86.7) | 69.7 (46.8 – 87.5) |
|  | 2010 | 258 | 21 | 11 | 52.4 (32.4 – 71.7) | 52.7 (32.3 – 72.3) |
|  | 2011 | 283 | 10 | 6 | 60.0 (31.3 – 83.2) | 59.3 (30.9 – 83.9) |
|  | 2012 | 216 | 10 | 8 | 80.0 (49.0 – 94.3) | 77.2 (48.6 – 94.9) |
|  | 2013 | 163 | 17 | 6 | 35.3 (17.3 – 58.7) | 36.6 (17.1 – 59.5) |
|  | 2015 | 177 | 9 | 6 | 66.7 (35.4 – 87.9) | 65.0 (34.9 – 88.6) |
|  | 2018 | 155 | 21 | 8 | 38.1 (20.8 – 59.1) | 39.1 (20.7 – 59.8) |
|  | 2020 | 270 | 6 | 3 | 50.0 (18.8 – 81.2) | 50.3 (18.4 – 82.1) |
|  | 2021 | 380 | 19 | 16 | 84.2 (62.4 – 94.5) | 82.7 (62.4 – 95.2) |
|  | 2022 | 320 | 25 | 25 | 100.0 (86.7 – 100.0) | 97.4 (89.0 – 100.0) |
|  | 2023 | 313 | 10 | 9 | 90.0 (59.6 – 99.5) | 86.0 (59.3 – 98.4) |
| **BoHV-1 gE** | 2011 | 283 | 10 | 5 | 50.0 (23.7 – 76.3) | 50.5 (23.6 – 77.4) |
|  | 2012 | 216 | 9 | 6 | 66.7 (35.4 – 87.9) | 65.2 (35.2 – 88.7) |
|  | 2013 | 163 | 8 | 5 | 62.5 (30.6 – 86.3) | 61.3 (30.4 – 87.2) |
|  | 2023 | 313 | 3 | 2 | 66.7 (20.8 – 98.3) | 61.9 (19.5 – 94.0) |
| **BTV** | 2007 | 379 | 20 | 0 | 0.0 (0.0 – 16.1) | 3.2 (0.0 – 13.2) |
|  | 2008 | 404 | 16 | 14 | 87.5 (64.0 – 96.5) | 84.6 (63.6 – 96.2) |
|  | 2009 | 311 | 17 | 13 | 76.5 (52.7 – 90.4) | 74.4 (52.2 – 90.3) |
|  | 2010 | 258 | 21 | 14 | 66.7 (45.4 – 82.8) | 65.6 (45.2 – 82.8) |
|  | 2011 | 283 | 20 | 9 | 45.0 (25.8 – 65.8) | 45.1 (25.6 – 65.9) |
|  | 2012 | 216 | 15 | 7 | 46.7 (24.8 – 69.9) | 46.9 (24.6 – 70.2) |
|  | 2013 | 163 | 19 | 7 | 36.8 (19.1 – 59.0) | 37.7 (18.9 – 59.1) |
|  | 2015 | 177 | 9 | 4 | 44.4 (18.9 – 73.3) | 45.2 (18.5 – 73.7) |
|  | 2018 | 155 | 21 | 6 | 28.6 (13.8 – 50.0) | 29.8 (13.8 – 50.2) |
|  | 2020 | 270 | 6 | 1 | 16.7 (0.9 – 56.4) | 22.7 (3.5 – 57.8) |
|  | 2021 | 380 | 19 | 1 | 5.3 (0.3 – 24.6) | 8.1 (1.1 – 24.7) |
|  | 2022 | 320 | 27 | 0 | 0.0 (0.0 – 12.5) | 2.4 (0.0 – 10.1) |
|  | 2023 | 313 | 10 | 2 | 20.0 (5.7 – 51.0) | 23.5 (5.9 – 51.9) |
| **BVDV ab** | 1998 | 434 | 15 | 0 | 0.0 (0.0 – 20.4) | 4.4 (0.0 – 17.7) |
|  | 1999 | 442 | 19 | 0 | 0.0 (0.0 – 16.8) | 3.5 (0.0 – 14.3) |
|  | 2000 | 500 | 19 | 0 | 0.0 (0.0 – 16.8) | 3.5 (0.0 – 14.2) |
|  | 2001 | 486 | 19 | 0 | 0.0 (0.0 – 16.8) | 3.5 (0.0 – 14.2) |
|  | 2002 | 539 | 14 | 0 | 0.0 (0.0 – 21.5) | 4.6 (0.0 – 18.4) |
|  | 2003 | 485 | 19 | 0 | 0.0 (0.0 – 16.8) | 3.5 (0.0 – 14.4) |
|  | 2004 | 573 | 8 | 0 | 0.0 (0.0 – 32.4) | 7.6 (0.0 – 28.9) |
|  | 2005 | 442 | 19 | 0 | 0.0 (0.0 – 16.8) | 3.5 (0.0 – 14.0) |
|  | 2006 | 379 | 15 | 0 | 0.0 (0.0 – 20.4) | 4.3 (0.0 – 17.4) |
|  | 2007 | 379 | 20 | 0 | 0.0 (0.0 – 16.1) | 3.3 (0.0 – 13.3) |
|  | 2008 | 404 | 12 | 0 | 0.0 (0.0 – 24.2) | 5.2 (0.0 – 20.8) |
|  | 2009 | 311 | 15 | 0 | 0.0 (0.0 – 20.4) | 4.3 (0.0 – 17.3) |
|  | 2010 | 258 | 21 | 2 | 9.5 (2.7 – 28.9) | 11.4 (2.0 – 29.1) |
|  | 2011 | 283 | 20 | 1 | 5.0 (0.3 – 23.6) | 7.2 (0.6 – 23.6) |
|  | 2012 | 216 | 18 | 0 | 0.0 (0.0 – 17.6) | 3.6 (0.0 – 15.0) |
|  | 2013 | 163 | 25 | 0 | 0.0 (0.0 – 13.3) | 2.7 (0.0 – 11.0) |
|  | 2015 | 177 | 9 | 2 | 22.2 (6.3 – 54.7) | 25.6 (5.9 – 56.0) |
|  | 2018 | 155 | 21 | 0 | 0.0 (0.0 – 15.5) | 3.2 (0.0 – 13.0) |
|  | 2020 | 270 | 6 | 0 | 0.0 (0.0 – 39.0) | 9.5 (0.0 – 35.6) |
|  | 2021 | 380 | 19 | 0 | 0.0 (0.0 – 16.8) | 3.5 (0.0 – 14.2) |
|  | 2022 | 320 | 25 | 0 | 0.0 (0.0 – 13.3) | 2.7 (0.0 – 11.1) |
|  | 2023 | 313 | 10 | 0 | 0.0 (0.0 – 27.8) | 6.2 (0.0 – 24.3) |
| **BVDV ag** | 1998 | 434 | 15 | 0 | 0.0 (0.0 – 20.4) | 4.3 (0.0 – 17.5) |
|  | 1999 | 442 | 18 | 0 | 0.0 (0.0 – 17.6) | 3.6 (0.0 – 14.7) |
|  | 2000 | 500 | 19 | 0 | 0.0 (0.0 – 16.8) | 3.4 (0.0 – 13.9) |
|  | 2001 | 486 | 16 | 0 | 0.0 (0.0 – 19.4) | 4.1 (0.0 – 16.5) |
|  | 2002 | 539 | 16 | 0 | 0.0 (0.0 – 19.4) | 4.1 (0.0 – 16.5) |
|  | 2003 | 485 | 19 | 1 | 5.3 (0.3 – 24.6) | 7.9 (0.9 – 24.8) |
|  | 2004 | 573 | 10 | 0 | 0.0 (0.0 – 27.8) | 6.2 (0.0 – 24.5) |
|  | 2005 | 442 | 18 | 0 | 0.0 (0.0 – 17.6) | 3.6 (0.0 – 14.9) |
|  | 2006 | 379 | 15 | 0 | 0.0 (0.0 – 20.4) | 4.3 (0.0 – 17.2) |
|  | 2007 | 379 | 20 | 0 | 0.0 (0.0 – 16.1) | 3.3 (0.0 – 13.6) |
|  | 2008 | 404 | 16 | 0 | 0.0 (0.0 – 19.4) | 4.1 (0.0 – 16.6) |
|  | 2009 | 311 | 16 | 2 | 12.5 (3.5 – 36.0) | 15.1 (3.4 – 36.2) |
|  | 2010 | 258 | 21 | 0 | 0.0 (0.0 – 15.5) | 3.1 (0.0 – 12.7) |
|  | 2011 | 283 | 20 | 1 | 5.0 (0.3 – 23.6) | 7.5 (0.8 – 23.9) |
|  | 2012 | 216 | 17 | 0 | 0.0 (0.0 – 18.4) | 3.9 (0.0 – 15.6) |
|  | 2013 | 163 | 23 | 0 | 0.0 (0.0 – 14.3) | 2.9 (0.0 – 11.8) |
|  | 2015 | 177 | 7 | 0 | 0.0 (0.0 – 35.4) | 8.4 (0.0 – 31.3) |
|  | 2018 | 155 | 21 | 0 | 0.0 (0.0 – 15.5) | 3.2 (0.0 – 12.9) |
|  | 2020 | 270 | 6 | 0 | 0.0 (0.0 – 39.0) | 9.5 (0.0 – 35.1) |
|  | 2021 | 380 | 19 | 0 | 0.0 (0.0 – 16.8) | 3.4 (0.0 – 14.0) |
|  | 2022 | 320 | 25 | 0 | 0.0 (0.0 – 13.3) | 2.7 (0.0 – 11.1) |
|  | 2023 | 313 | 10 | 0 | 0.0 (0.0 – 27.8) | 6.2 (0.0 – 24.3) |
| ***C. burnetii*** | 2010 | 258 | 18 | 0 | 0.0 (0.0 – 17.6) | 4.0 (0.0 – 16.0) |
|  | 2011 | 283 | 20 | 0 | 0.0 (0.0 – 16.1) | 3.6 (0.0 – 14.8) |
|  | 2012***** | 216 | 15 | 1 | 6.7 (0.3 – 41.4) | 13.8 (0.9 – 43.8) |
|  | 2013***** | 163 | 24 | 1 | 4.2 (0.2 – 29.4) | 8.7 (0.4 – 30.4) |
|  | 2015 | 177 | 9 | 0 | 0.0 (0.0 – 29.9) | 7.5 (0.0 – 28.9) |
|  | 2018***** | 155 | 19 | 0 | 0.0 (0.0 – 28.4) | 7.0 (0.0 – 26.9) |
|  | 2020 | 270 | 6 | 0 | 0.0 (0.0 – 39.0) | 10.5 (0.0 – 38.8) |
|  | 2021***** | 380 | 19 | 0 | 0.0 (0.0 – 28.6) | 7.0 (0.0 – 27.2) |
|  | 2022***** | 320 | 26 | 0 | 0.0 (0.0 – 22.6) | 5.3 (0.0 – 20.9) |
|  | 2023***** | 313 | 10 | 0 | 0.0 (0.0 – 41.6) | 11.4 (0.0 – 41.1) |
| ***F. hepatica*** | 1998 | 434 | 9 | 0 | 0.0 (0.0 – 29.9) | 13.4 (0.0 – 51.6) |
|  | 1999 | 442 | 18 | 1 | 5.6 (0.3 – 25.8) | 9.5 (0.4 – 43.4) |
|  | 2000 | 500 | 18 | 3 | 16.7 (5.8 – 39.2) | 18.4 (0.9 – 62.6) |
|  | 2001 | 486 | 14 | 0 | 0.0 (0.0 – 21.5) | 9.0 (0.0 – 36.0) |
|  | 2002 | 539 | 15 | 8 | 53.3 (30.1 – 75.2) | 76.5 (31.3 – 98.8) |
|  | 2003 | 485 | 20 | 3 | 15.0 (5.2 – 36.0) | 15.7 (0.7 – 56.4) |
|  | 2004 | 573 | 7 | 0 | 0.0 (0.0 – 35.4) | 16.9 (0.0 – 62.2) |
|  | 2005 | 442 | 18 | 3 | 16.7 (5.8 – 39.2) | 18.5 (0.9 – 62.9) |
|  | 2006 | 379 | 12 | 3 | 25.0 (8.9 – 53.2) | 33.6 (2.3 – 87.0) |
|  | 2007 | 379 | 17 | 11 | 64.7 (41.3 – 82.7) | 63.4 (40.4 – 83.0) |
|  | 2008 | 404 | 14 | 14 | 100.0 (78.5 – 100.0) | 95.4 (81.6 – 100.0) |
|  | 2009 | 311 | 13 | 13 | 100.0 (77.2 – 100.0) | 95.1 (80.3 – 100.0) |
|  | 2010 | 258 | 21 | 20 | 95.2 (77.3 – 99.8) | 92.9 (77.3 – 99.3) |
|  | 2011 | 283 | 19 | 13 | 68.4 (46.0 – 84.6) | 67.1 (45.3 – 84.8) |
|  | 2012 | 216 | 19 | 16 | 84.2 (62.4 – 94.5) | 82.1 (62.0 – 94.7) |
|  | 2013 | 163 | 22 | 22 | 100.0 (85.1 – 100.0) | 97.0 (87.7 – 100.0) |
|  | 2015 | 177 | 9 | 9 | 100.0 (70.1 – 100.0) | 93.3 (73.7 – 100.0) |
|  | 2018 | 155 | 21 | 18 | 85.7 (65.4 – 95.0) | 83.8 (65.0 – 95.3) |
|  | 2020 | 270 | 6 | 4 | 66.7 (30.0 – 90.3) | 63.4 (28.5 – 90.4) |
|  | 2021 | 380 | 18 | 7 | 38.9 (20.3 – 61.4) | 39.0 (19.0 – 61.3) |
|  | 2022 | 320 | 26 | 16 | 61.5 (42.5 – 77.6) | 60.7 (41.7 – 77.6) |
|  | 2023 | 313 | 5 | 4 | 80.0 (37.6 – 99.0) | 73.6 (35.3 – 96.1) |
| ***MAP*** | 1998 | 434 | 15 | 0 | 0.0 (0.0 – 20.4) | *Na* |
|  | 1999***** | 442 | 32 | 8 | 25.0 (9.2 – 60.6) | *Na* |
|  | 2000***** | 500 | 19 | 1 | 5.3 (0.3 – 39.9) | *Na* |
|  | 2001***** | 486 | 19 | 0 | 0.0 (0.0 – 33.3) | *Na* |
|  | 2002***** | 539 | 18 | 1 | 5.6 (0.3 – 59.0) | *Na* |
|  | 2003***** | 485 | 21 | 0 | 0.0 (0.0 – 24.3) | *Na* |
|  | 2004***** | 573 | 17 | 0 | 0.0 (0.0 – 45.9) | *Na* |
|  | 2005***** | 442 | 21 | 0 | 0.0 (0.0 – 34.5) | *Na* |
|  | 2006***** | 379 | 20 | 0 | 0.0 (0.0 – 27.2) | *Na* |
|  | 2007 | 379 | 20 | 3 | 15.0 (5.2 – 36.0) | *Na* |
|  | 2008***** | 404 | 19 | 0 | 0.0 (0.0 – 25.2) | *Na* |
|  | 2009***** | 311 | 17 | 1 | 5.9 (0.3 – 44.8) | *Na* |
|  | 2010***** | 258 | 21 | 0 | 0.0 (0.0 – 19.9) | *Na* |
|  | 2011***** | 283 | 18 | 0 | 0.0 (0.0 – 29.8) | *Na* |
|  | 2012 | 216 | 10 | 2 | 20.0 (5.7 – 51.0) | *Na* |
|  | 2013***** | 163 | 17 | 1 | 5.9 (0.3 – 38.2) | *Na* |
|  | 2015***** | 177 | 9 | 1 | 11.1 (0.6 – 52.4) | *Na* |
|  | 2018***** | 155 | 21 | 0 | 0.0 (0.0 – 25.7) | *Na* |
|  | 2020 | 270 | 6 | 1 | 16.7 (0.9 – 56.4) | *Na* |
|  | 2021***** | 380 | 20 | 1 | 5.0 (0.3 – 38.5) | *Na* |
|  | 2022***** | 320 | 27 | 1 | 3.7 (0.2 – 26.6) | *Na* |
|  | 2023***** | 313 | 12 | 0 | 0.0 (0.0 – 47.2) | *Na* |
| ***S.* Dublin** | 2002 | 539 | 10 | 2 | 20.0 (5.7 – 51.0) | 25.8 (2.4 – 67.5) |
|  | 2003 | 485 | 19 | 0 | 0.0 (0.0 – 16.8) | 4.7 (0.0 – 19.3) |
|  | 2004 | 573 | 4 | 0 | 0.0 (0.0 – 49.0) | 17.9 (0.0 – 62.7) |
|  | 2005 | 442 | 20 | 0 | 0.0 (0.0 – 16.1) | 4.5 (0.0 – 18.5) |
|  | 2006 | 379 | 13 | 1 | 7.7 (0.4 – 33.3) | 11.6 (0.5 – 43.0) |
|  | 2007 | 379 | 20 | 1 | 5.0 (0.3 – 23.6) | 7.0 (0.3 – 28.8) |
|  | 2008 | 404 | 12 | 3 | 25.0 (8.9 – 53.2) | 31.2 (4.7 – 69.8) |
|  | 2009 | 311 | 15 | 0 | 0.0 (0.0 – 20.4) | 5.9 (0.0 – 23.7) |
|  | 2010 | 258 | 21 | 4 | 19.0 (7.7 – 40.0) | 21.4 (3.0 – 50.3) |
|  | 2011 | 283 | 10 | 0 | 0.0 (0.0 – 27.8) | 8.3 (0.0 – 33.0) |
|  | 2012 | 216 | 15 | 0 | 0.0 (0.0 – 20.4) | 5.9 (0.0 – 24.1) |
|  | 2013 | 163 | 18 | 2 | 11.1 (3.1 – 32.8) | 12.6 (0.7 – 40.4) |
|  | 2015 | 177 | 9 | 1 | 11.1 (0.6 – 43.5) | 17.3 (0.9 – 58.1) |
|  | 2018 | 155 | 21 | 2 | 9.5 (2.7 – 28.9) | 10.1 (0.5 – 34.6) |
|  | 2020 | 270 | 6 | 0 | 0.0 (0.0 – 39.0) | 13.2 (0.0 – 48.7) |
|  | 2021 | 380 | 18 | 0 | 0.0 (0.0 – 17.6) | 5.0 (0.0 – 20.4) |
|  | 2022 | 320 | 24 | 0 | 0.0 (0.0 – 13.8) | 3.8 (0.0 – 15.7) |
|  | 2023 | 313 | 9 | 2 | 22.2 (6.3 – 54.7) | 28.9 (3.0 – 72.9) |
| ***Salmonella* spp.** | 1998 | 434 | 15 | 0 | 0.0 (0.0 – 20.4) | 20.1 (0.0 – 73.8) |
|  | 1999 | 442 | 20 | 0 | 0.0 (0.0 – 16.1) | 16.0 (0.0 – 62.5) |
|  | 2000 | 500 | 20 | 0 | 0.0 (0.0 – 16.1) | 15.8 (0.0 – 63.1) |
|  | 2001 | 486 | 19 | 0 | 0.0 (0.0 – 16.8) | 16.6 (0.0 – 64.6) |
|  | 2002 | 539 | 18 | 0 | 0.0 (0.0 – 17.6) | 17.3 (0.0 – 66.8) |
|  | 2003 | 485 | 21 | 0 | 0.0 (0.0 – 15.5) | 15.4 (0.0 – 60.7) |
|  | 2004 | 573 | 17 | 0 | 0.0 (0.0 – 18.4) | 18.5 (0.0 – 69.8) |
|  | 2005 | 442 | 18 | 0 | 0.0 (0.0 – 17.6) | 17.6 (0.0 – 67.5) |
|  | 2006 | 379 | 20 | 0 | 0.0 (0.0 – 16.1) | 16.0 (0.0 – 63.5) |
|  | 2007 | 379 | 20 | 0 | 0.0 (0.0 – 16.1) | 15.9 (0.0 – 63.1) |
|  | 2008 | 404 | 19 | 0 | 0.0 (0.0 – 16.8) | 16.7 (0.0 – 64.8) |
|  | 2009 | 311 | 17 | 0 | 0.0 (0.0 – 18.4) | 18.1 (0.0 – 69.3) |
|  | 2010 | 258 | 21 | 0 | 0.0 (0.0 – 15.5) | 15.4 (0.0 – 61.1) |
|  | 2011***** | 283 | 20 | 0 | 0.0 (0.0 – 27.8) | 16.8 (0.0 – 54.9) |
|  | 2012***** | 216 | 14 | 1 | 7.1 (0.4 – 40.4) | 27.3 (1.6 – 75.8) |
|  | 2013***** | 163 | 17 | 1 | 5.9 (0.3 – 36.6) | 24.9 (1.2 – 75.1) |
|  | 2015 | 177 | 9 | 0 | 0.0 (0.0 – 29.9) | 28.7 (0.0 – 86.2) |
|  | 2018 | 155 | 6 | 0 | 0.0 (0.0 – 39.0) | 34.5 (0.0 – 90.1) |
|  | 2020 | 270 | 6 | 0 | 0.0 (0.0 – 39.0) | 34.5 (0.0 – 89.7) |
|  | 2021 | 380 | 12 | 0 | 0.0 (0.0 – 24.2) | 23.9 (0.0 – 80.4) |
|  | 2022***** | 320 | 12 | 0 | 0.0 (0.0 – 34.1) | 27.5 (0.0 – 84.0) |
|  | 2023***** | 313 | 12 | 0 | 0.0 (0.0 – 36.5) | 25.5 (0.0 – 78.3) |
| **SBV** | 2015 | 177 | 9 | 6 | 66.7 (35.4 – 87.9) | 63.8 (32.3 – 88.4) |
|  | 2018 | 155 | 16 | 15 | 93.8 (71.7 – 99.7) | 90.9 (71.0 – 99.2) |
|  | 2020 | 270 | 6 | 4 | 66.7 (30.0 – 90.3) | 62.9 (26.5 – 90.7) |
|  | 2021 | 380 | 19 | 19 | 100.0 (83.2 – 100.0) | 96.5 (85.7 – 100.0) |
|  | 2022 | 320 | 26 | 21 | 80.8 (62.1 – 91.5) | 79.4 (61.1 – 92.2) |
|  | 2023 | 313 | 10 | 6 | 60.0 (31.3 – 83.2) | 57.9 (28.1 – 83.5) |
| ***** weighted estimates; *Na* not assessed. | | | | | | |

Table S4 – Apparent prevalence (AP) with Wilson’s 95% confidence interval (CI) and median true prevalence (TP) with 95% uncertainty interval (UI) per pathogen per year for red deer. *N* indicates total population, *n* sample population and *x* number of positive samples.

| **Pathogen** | **Year** | **N** | **n** | **x** | **AP (CI) %** | **TP (UI) %** |
| --- | --- | --- | --- | --- | --- | --- |
| **BoHV-1 gB** | 2007 | 1758 | 8 | 0 | 0.0 (0.0 – 32.4) | 7.5 (0.0 – 28.4) |
|  | 2011 | 2454 | 10 | 4 | 40.0 (16.8 – 68.7) | 41.5 (16.6 – 69.8) |
|  | 2012 | 2311 | 3 | 0 | 0.0 (0.0 – 56.1) | 16.2 (0.0 – 53.4) |
|  | 2013 | 2055 | 12 | 0 | 0.0 (0.0 – 24.2) | 5.2 (0.0 – 20.8) |
|  | 2015 | 2229 | 6 | 4 | 66.7 (30.0 – 90.3) | 64.1 (29.5 – 90.9) |
|  | 2016 | 1778 | 5 | 3 | 60.0 (23.1 – 88.2) | 58.3 (22.3 – 89.0) |
|  | 2017 | 2650 | 13 | 3 | 23.1 (8.2 – 50.3) | 25.5 (8.2 – 50.9) |
|  | 2018 | 1275 | 16 | 15 | 93.8 (71.7 – 99.7) | 91.0 (71.8 – 99.2) |
|  | 2019 | 1100 | 15 | 11 | 73.3 (48.0 – 89.1) | 72.2 (48.2 – 89.9) |
|  | 2020 | 1600 | 21 | 8 | 38.1 (20.8 – 59.1) | 39.0 (20.6 – 59.9) |
|  | 2021 | 1870 | 15 | 9 | 60.0 (35.7 – 80.2) | 59.7 (35.5 – 81.1) |
|  | 2022 | 1625 | 11 | 8 | 72.7 (43.4 – 90.3) | 70.9 (43.2 – 91.1) |
|  | 2023 | 700 | 6 | 2 | 33.3 (9.7 – 70.0) | 36.6 (9.7 – 71.8) |
| **BoHV-1 gE** | 2011 | 2454 | 4 | 1 | 25.0 (1.3 – 69.9) | 31.8 (5.3 – 72.5) |
|  | 2012 | 2311 | 7 | 1 | 14.3 (0.7 – 51.3) | 20.3 (3.2 – 53.1) |
|  | 2013 | 2055 | 10 | 2 | 20.0 (5.7 – 51.0) | 23.9 (6.0 – 52.3) |
| **BTV** | 2011 | 2454 | 14 | 4 | 28.6 (11.7 – 54.6) | 30.3 (11.7 – 55.0) |
|  | 2012 | 2311 | 10 | 0 | 0.0 (0.0 – 27.8) | 6.1 (0.0 – 23.6) |
|  | 2013 | 2055 | 21 | 0 | 0.0 (0.0 – 15.5) | 3.1 (0.0 – 12.4) |
|  | 2015 | 2229 | 6 | 0 | 0.0 (0.0 – 39.0) | 9.4 (0.0 – 34.6) |
|  | 2016 | 1778 | 5 | 2 | 40.0 (11.8 – 76.9) | 42.1 (11.7 – 77.5) |
|  | 2017 | 2650 | 13 | 0 | 0.0 (0.0 – 22.8) | 4.9 (0.0 – 19.3) |
|  | 2018 | 1275 | 14 | 2 | 14.3 (4.0 – 39.9) | 17.4 (4.2 – 40.2) |
|  | 2019 | 1100 | 15 | 1 | 6.7 (0.3 – 29.8) | 10.1 (1.5 – 30.2) |
|  | 2020 | 1600 | 21 | 0 | 0.0 (0.0 – 15.5) | 3.1 (0.0 – 12.7) |
|  | 2021 | 1870 | 14 | 0 | 0.0 (0.0 – 21.5) | 4.5 (0.0 – 18.2) |
|  | 2022 | 1625 | 10 | 1 | 10.0 (0.5 – 40.4) | 14.8 (2.2 – 41.0) |
|  | 2023 | 700 | 6 | 0 | 0.0 (0.0 – 39.0) | 9.5 (0.0 – 35.0) |
| ***F. hepatica*** | 2011 | 2454 | 14 | 0 | 0.0 (0.0 – 21.5) | 4.6 (0.0 – 18.3) |
|  | 2012 | 2311 | 10 | 0 | 0.0 (0.0 – 27.8) | 6.2 (0.0 – 23.8) |
|  | 2013 | 2055 | 22 | 0 | 0.0 (0.0 – 14.9) | 3.0 (0.0 – 12.4) |
|  | 2016 | 1778 | 3 | 1 | 33.3 (1.7 – 79.2) | 38.0 (5.6 – 80.8) |
|  | 2017 | 2650 | 8 | 3 | 37.5 (13.7 – 69.4) | 38.7 (12.6 – 70.0) |
|  | 2018 | 1275 | 7 | 2 | 28.6 (8.2 – 64.1) | 31.4 (7.1 – 64.9) |
|  | 2019 | 1100 | 7 | 1 | 14.3 (0.7 – 51.3) | 19.2 (2.1 – 52.1) |
|  | 2020 | 1600 | 7 | 0 | 0.0 (0.0 – 35.4) | 8.4 (0.0 – 31.6) |
|  | 2021 | 1870 | 9 | 0 | 0.0 (0.0 – 29.9) | 6.8 (0.0 – 26.0) |
|  | 2023 | 700 | 4 | 0 | 0.0 (0.0 – 49.0) | 13.0 (0.0 – 45.4) |
| ***MAP*** | 2007***** | 1758 | 10 | 0 | 0.0 (0.0 – 41.6) | *Na* |
|  | 2011 | 2454 | 10 | 2 | 20.0 (5.7 – 51.0) | *Na* |
|  | 2012 | 2311 | 3 | 0 | 0.0 (0.0 – 56.1) | *Na* |
|  | 2013***** | 2055 | 12 | 0 | 0.0 (0.0 – 36.5) | *Na* |
|  | 2015 | 2229 | 6 | 0 | 0.0 (0.0 – 39.0) | *Na* |
|  | 2018 | 1275 | 6 | 1 | 16.7 (0.9 – 56.4) | *Na* |
|  | 2019***** | 1100 | 13 | 7 | 53.8 (25.6 – 81.2) | *Na* |
|  | 2020***** | 1600 | 26 | 4 | 15.4 (6.7 – 35.0) | *Na* |
|  | 2021***** | 1870 | 10 | 3 | 30.0 (11.5 – 65.8) | *Na* |
|  | 2022***** | 1625 | 10 | 1 | 10.0 (0.5 – 56.6) | *Na* |
|  | 2023***** | 700 | 5 | 1 | 20.0 (1.0 – 84.3) | *Na* |
| ***S.* Dublin** | 2011 | 2454 | 14 | 0 | 0.0 (0.0 – 21.5) | 6.2 (0.0 – 24.9) |
|  | 2012 | 2311 | 7 | 0 | 0.0 (0.0 – 35.4) | 11.5 (0.0 – 43.2) |
|  | 2013 | 2055 | 22 | 0 | 0.0 (0.0 – 14.9) | 4.1 (0.0 – 16.9) |
|  | 2016 | 1778 | 5 | 0 | 0.0 (0.0 – 43.4) | 15.1 (0.0 – 54.0) |
|  | 2017 | 2650 | 12 | 2 | 16.7 (4.7 – 44.8) | 20.8 (1.6 – 58.3) |
|  | 2018 | 1275 | 11 | 1 | 9.1 (0.5 – 37.7) | 13.6 (0.7 – 49.2) |
|  | 2019 | 1100 | 10 | 0 | 0.0 (0.0 – 27.8) | 8.5 (0.0 – 32.9) |
|  | 2020 | 1600 | 9 | 0 | 0.0 (0.0 – 29.9) | 9.4 (0.0 – 36.5) |
|  | 2021 | 1870 | 10 | 0 | 0.0 (0.0 – 27.8) | 8.4 (0.0 – 33.1) |
|  | 2022 | 1625 | 3 | 0 | 0.0 (0.0 – 56.1) | 22.0 (0.0 – 71.3) |
|  | 2023 | 700 | 4 | 1 | 25.0 (1.3 – 69.9) | 37.4 (2.8 – 90.1) |
| ***S.* Typhimurium** | 2011 | 2454 | 14 | 0 | 0.0 (0.0 – 21.5) | 6.4 (0.0 – 25.4) |
|  | 2012 | 2311 | 7 | 2 | 28.6 (8.2 – 64.1) | 38.1 (4.9 – 85.2) |
|  | 2013 | 2055 | 22 | 0 | 0.0 (0.0 – 14.9) | 4.1 (0.0 – 17.0) |
|  | 2016 | 1778 | 5 | 0 | 0.0 (0.0 – 43.4) | 15.1 (0.0 – 54.7) |
|  | 2017 | 2650 | 12 | 0 | 0.0 (0.0 – 24.2) | 7.2 (0.0 – 28.5) |
|  | 2018 | 1275 | 11 | 1 | 9.1 (0.5 – 37.7) | 13.9 (0.7 – 48.8) |
|  | 2019 | 1100 | 10 | 0 | 0.0 (0.0 – 27.8) | 8.6 (0.0 – 33.3) |
|  | 2020 | 1600 | 9 | 0 | 0.0 (0.0 – 29.9) | 9.2 (0.0 – 35.5) |
|  | 2021 | 1870 | 9 | 2 | 22.2 (6.3 – 54.7) | 29.0 (3.0 – 73.0) |
|  | 2022 | 1625 | 3 | 0 | 0.0 (0.0 – 56.1) | 21.8 (0.0 – 71.1) |
|  | 2023 | 700 | 4 | 0 | 0.0 (0.0 – 49.0) | 17.9 (0.0 – 62.1) |
| *****weighted estimates; *Na* not assessed. | | | | | | |

Table S5 – Apparent prevalence (AP) with Wilson’s 95% confidence interval (CI) and median true prevalence (TP) with 95% uncertainty interval (UI) per pathogen per year for Konik horses. *N* indicates total population, *n* sample population and *x* number of positive samples.

| **Pathogen** | **Year** | **N** | **n** | **x** | **AP (CI) %** | **TP (UI) %** |
| --- | --- | --- | --- | --- | --- | --- |
| **EHV-1** | 2012 | 808 | 6 | 1 | 16.7 (0.9 – 56.4) | 22.1 (2.8 – 57.7) |
|  | 2016 | 758 | 6 | 0 | 0.0 (0.0 – 39.0) | 9.4 (0.0 – 34.9) |
|  | 2017 | 865 | 6 | 0 | 0.0 (0.0 – 39.0) | 9.4 (0.0 – 34.8) |
|  | 2018 | 580 | 6 | 0 | 0.0 (0.0 – 39.0) | 9.4 (0.0 – 34.7) |
| **EHV-4** | 2012 | 808 | 6 | 0 | 0.0 (0.0 – 39.0) | 9.5 (0.0 – 34.5) |
|  | 2016 | 758 | 6 | 0 | 0.0 (0.0 – 39.0) | 9.4 (0.0 – 35.1) |
|  | 2017 | 865 | 6 | 2 | 33.3 (9.7 – 70.0) | 35.5 (8.9 – 70.8) |
|  | 2018 | 580 | 6 | 0 | 0.0 (0.0 – 39.0) | 9.5 (0.0 – 34.8) |
| ***S.* Dublin** | 2011 | 867 | 3 | 0 | 0.0 (0.0 – 56.1) | 22.0 (0.0 – 71.0) |
|  | 2013 | 818 | 12 | 0 | 0.0 (0.0 – 24.2) | 7.2 (0.0 – 28.9) |
|  | 2015 | 1006 | 10 | 0 | 0.0 (0.0 – 27.8) | 8.6 (0.0 – 33.2) |
|  | 2016 | 758 | 8 | 3 | 37.5 (13.7 – 69.4) | 48.3 (10.6 – 90.4) |
|  | 2018 | 580 | 9 | 2 | 22.2 (6.3 – 54.7) | 29.1 (2.9 – 73.0) |
|  | 2019 | 380 | 3 | 1 | 33.3 (1.7 – 79.2) | 45.9 (4.1 – 94.8) |
|  | 2020 | 480 | 8 | 6 | 75.0 (40.9 – 92.9) | 83.2 (44.2 – 99.2) |
|  | 2021 | 280 | 7 | 0 | 0.0 (0.0 – 35.4) | 11.6 (0.0 – 43.8) |
|  | 2022 | 280 | 5 | 0 | 0.0 (0.0 – 43.4) | 15.0 (0.0 – 54.5) |
|  | 2023 | 315 | 4 | 1 | 25.0 (1.3 – 69.9) | 37.4 (2.9 – 90.2) |
| ***S.* Typhimurium** | 2011 | 867 | 3 | 0 | 0.0 (0.0 – 56.1) | 22.1 (0.0 – 71.9) |
|  | 2013 | 818 | 12 | 0 | 0.0 (0.0 – 24.2) | 7.2 (0.0 – 28.5) |
|  | 2015 | 1006 | 10 | 1 | 10.0 (0.5 – 40.4) | 15.5 (0.8 – 52.8) |
|  | 2016 | 758 | 8 | 0 | 0.0 (0.0 – 32.4) | 10.2 (0.0 – 38.7) |
|  | 2018 | 580 | 9 | 0 | 0.0 (0.0 – 29.9) | 9.3 (0.0 – 36.2) |
|  | 2019 | 380 | 3 | 0 | 0.0 (0.0 – 56.1) | 22.0 (0.0 – 71.4) |
|  | 2020 | 480 | 8 | 2 | 25.0 (7.1 – 59.1) | 33.0 (3.7 – 79.2) |
|  | 2021 | 280 | 7 | 0 | 0.0 (0.0 – 35.4) | 11.5 (0.0 – 43.8) |
|  | 2022 | 280 | 5 | 0 | 0.0 (0.0 – 43.4) | 15.1 (0.0 – 54.7) |
|  | 2023 | 315 | 4 | 1 | 25.0 (1.3 – 69.9) | 37.5 (2.9 – 90.1) |
| ***Salmonella* spp.** | 2007 | 763 | 7 | 0 | 0.0 (0.0 – 35.4) | 32.6 (0.0 – 88.8) |
|  | 2011***** | 867 | 11 | 0 | 0.0 (0.0 – 41.0) | 23.6 (0.0 – 66.4) |
|  | 2012***** | 808 | 10 | 0 | 0.0 (0.0 – 43.4) | 15.0 (0.0 – 53.9) |
|  | 2013 | 818 | 10 | 2 | 20.0 (5.7 – 51.0) | 33.7 (8.6 – 74.0) |
|  | 2015 | 1006 | 10 | 0 | 0.0 (0.0 – 27.8) | 8.7 (0.0 – 33.8) |
|  | 2018 | 580 | 3 | 0 | 0.0 (0.0 – 56.1) | 22.7 (0.0 – 73.5) |
|  | 2019***** | 380 | 3 | 1 | 33.3 (1.7 – 77.2) | 42.5 (7.4 – 88.4) |
|  | 2020***** | 480 | 15 | 1 | 6.7 (0.3 – 32.8) | 13.1 (1.5 – 36.3) |
|  | 2021***** | 280 | 9 | 0 | 0.0 (0.0 – 42.2) | 19.1 (0.0 – 55.5) |
|  | 2022 | 280 | 8 | 0 | 0.0 (0.0 – 32.4) | 10.5 (0.0 – 40.6) |
|  | 2023 | 315 | 9 | 1 | 11.1 (0.6 – 39.9) | 17.1 (2.5 – 46.8) |
| ***** weighted estimates. | | | | | | |

## Parasite results

Table S6 – Overview of Heck cattle parasite results, including number of samples tested per year and respective proportion of positive samples, and median eggs per gram of faeces (EPG).

| Year | Ectoparasites | *Eimeria* spp. | *M. expansa* | Lungworm* | *Nematodirus* spp. | *N. battus* | Trichostrongylus/Strongylus eggs | *S. papillosus* | Other endoparasites* |
| --- | --- | --- | --- | --- | --- | --- | --- | --- | --- |
| 1997 |  |  |  | 0/1 (0) |  |  |  |  |  |
| 1998 | 1/1 (100) |  |  | 0/15 (0) |  |  |  |  |  |
| 1999 |  | 7/29 (24.1) | 0/29 (0) | 0/7 (0) | 1/29 (3.4)  m: 50 | 0/29 (0) | 14/29 (48.3)  m: 150 | 3/29 (10.3)  m: 100 | 0/27 (0) |
| 2000 |  | 6/30 (20) | 0/31 (0) | 2/2 (100) | 0/31 (0) | 0/31 (0) | 7/31 (22.6)  m: 100 | 1/31 (3.2)  m: 50 | 0/30 (0) |
| 2001 |  | 8/28 (28.6) | 0/28 (0) |  | 0/28 (0) | 0/28 (0) | 13/28 (46.4)  m: 50 | 1/28 (3.6)  m: 50 | 1/28 (3.6) |
| 2002 | 1/1 (100) | 8/28 (28.6) | 0/28 (0) |  | 0/28 (0) | 0/28 (0) | 11/28 (39.3)  m: 100 | 5/28 (17.9)  m: 100 | 0/28 (0) |
| 2003 |  | 16/33 (48.5) | 0/33 (0) | 3/3 (100) | 1/33 (3)  m: 100 | 0/33 (0) | 17/33 (51.5)  m: 150 | 0/33 (0) | 0/33 (0) |
| 2004 |  | 12/32 (37.5) | 0/32 (0) |  | 0/32 (0) | 0/32 (0) | 9/32 (28.1)  m: 100 | 1/32 (3.1)  m: 100 | 0/32 (0) |
| 2005 |  | 13/23 (56.5) | 0/23 (0) |  | 2/23 (8.7)  m: 100 | 0/23 (0) | 15/23 (65.2)  m: 200 | 0/23 (0) | 0/23 (0) |
| 2006 |  | 9/30 (30) | 0/30 (0) |  | 1/30 (3.3)  m: 50 | 0/30 (0) | 14/30 (46.7)  m: 100 | 3/30 (10)  m: 50 | 0/29 (0) |
| 2007 |  | 10/30 (33.3) | 0/30 (0) |  | 0/30 (0) | 0/30 (0) | 12/30 (40)  m: 75 | 0/30 (0) | 1/30 (3.3) |
| 2008 |  | 8/19 (42.1) | 0/19 (0) |  | 0/19 (0) | 0/19 (0) | 6/19 (31.6)  m: 150 | 1/19 (5.3)  m: 50 | 1/19 (5.3) |
| 2009 |  | 5/17 (29.4) | 0/17 (0) |  | 0/17 (0) | 0/17 (0) | 8/17 (47.1)  m: 100 | 1/17 (5.9)  m: 50 | 0/16 (0) |
| 2010 |  | 8/21 (38.1) | 1/21 (4.8) |  | 0/21 (0) | 0/21 (0) | 7/21 (33.3)  m: 150 | 2/21 (9.5)  m: 125 | 0/21 (0) |
| 2011 |  | 7/18 (38.9) | 0/18 (0) |  | 0/18 (0) | 0/18 (0) | 5/18 (27.8)  m: 50 | 2/18 (11.1)  m: 50 | 0/17 (0) |
| 2012 |  | 1/10 (10) | 0/10 (0) |  | 0/10 (0) | 0/10 (0) | 3/10 (30)  m: 100 | 2/10 (20)  m: 125 | 0/10 (0) |
| 2013 |  | 0/5 (0) | 0/11 (0) |  | 1/11 (9.1)  m: 50 | 0/11 (0) | 1/11 (9.1)  m: 150 | 0/11 (0) | 0/11 (0) |
| 2015 |  | 0/9 (0) | 0/9 (0) |  | 0/9 (0) | 0/9 (0) | 3/9 (33.3)  m: 100 | 1/9 (11.1)  m: 50 | 0/9 (0) |
| 2017 |  | 0/2 (0) | 0/2 (0) |  | 0/2 (0) | 0/2 (0) | 0/2 (0) | 0/2 (0) | 0/2 (0) |
| 2018 |  | 0/6 (0) | 0/6 (0) |  | 0/6 (0) | 0/6 (0) | 0/6 (0) | 0/6 (0) | 0/6 (0) |
| 2019 |  | 0/1 (0) | 0/1 (0) |  | 0/1 (0) | 0/1 (0) | 0/1 (0) | 0/1 (0) | 0/1 (0) |
| 2020 |  | 2/5 (40) | 2/7 (28.6) |  | 0/7 (0) | 0/7 (0) | 1/7 (14.3)  m: 150 | 0/7 (0) | 0/7 (0) |
| 2021 |  | 3/12 (25) | 0/12 (0) |  | 0/12 (0) | 0/12 (0) | 0/12 (0) | 1/12 (8.3)  m: 50 | 0/12 (0) |
| 2022 | 0/5 (0) | 2/10 (20) | 0/12 (0) |  | 0/12 (0) | 0/12 (0) | 3/12 (25)  m: 50 | 1/12 (8.3)  m: 50 | 0/12 (0) |
| 2023 | 0/9 (0) | 0/9 (0) | 1/9 (11.1) |  | 0/9 (0) | 0/9 (0) | 4/9 (44.4)  m: 50 | 0/9 (0) | 0/9 (0) |
| Results read as: positive samples/number of samples (proportion of positive samples %), m: median of EPG (eggs per gram of faeces), empty cells indicate not tested. *Lungworm was identified as *Dictyocaulus viviparus*; other parasites detected were all *Capillaria* sp. | | | | | | | | | |

Table S7 – Overview of red deer parasite results, including number of samples tested per year and respective proportion of positive samples, and median eggs per gram of faeces (EPG).

| Year | Ectoparasites | *Eimeria* spp. | *M. expansa* | *Nematodirus* spp. | *N. battus* | Trichostrongylus/Strongylus eggs | *S. papillosus* | Other endoparasites* |
| --- | --- | --- | --- | --- | --- | --- | --- | --- |
| 2000 |  | 0/2 (0) | 0/2 (0) | 0/2 (0) | 0/2 (0) | 1/2 (50)  m: 100 | 0/2 (0) | 0/2 (0) |
| 2001 |  | 1/2 (50) | 0/2 (0) | 0/2 (0) | 0/2 (0) | 1/2 (50)  m: 100 | 0/2 (0) | 0/2 (0) |
| 2002 |  | 0/1 (0) | 0/1 (0) | 0/1 (0) | 0/1 (0) | 0/1 (0) | 0/1 (0) | 0/1 (0) |
| 2003 |  | 0/3 (0) | 0/3 (0) | 0/3 (0) | 0/3 (0) | 1/3 (33.3)  m: 300 | 0/3 (0) | 0/3 (0) |
| 2005 |  | 2/2 (100) | 0/2 (0) | 0/2 (0) | 0/2 (0) | 1/2 (50)  m: 50 | 0/2 (0) | 0/2 (0) |
| 2007 |  | 1/8 (12.5) | 0/8 (0) | 0/8 (0) | 0/8 (0) | 7/8 (87.5)  m: 150 | 0/8 (0) | 0/8 (0) |
| 2011 |  | 1/10 (10) | 0/10 (0) | 0/10 (0) | 0/10 (0) | 7/10 (70)  m: 100 | 0/10 (0) | 0/10 (0) |
| 2012 |  | 2/3 (66.7) | 0/3 (0) | 0/3 (0) | 0/3 (0) | 1/3 (33.3)  m: 150 | 0/3 (0) | 0/3 (0) |
| 2013 |  | 6/12 (50) | 0/3 (0) | 0/3 (0) | 0/3 (0) | 6/12 (50)  m: 95 | 1/3 (33.3)  m: 50 | 6/12 (50) |
| 2015 |  | 3/6 (50) | 0/6 (0) | 0/6 (0) | 0/6 (0) | 3/6 (50)  m: 100 | 0/6 (0) | 0/6 (0) |
| 2017 |  | 0/1 (0) | 0/1 (0) | 0/1 (0) | 0/1 (0) | 1/1 (100)  m: 50 | 0/1 (0) | 0/1 (0) |
| 2018 |  | 2/6 (33.3) | 0/6 (0) | 0/6 (0) | 0/6 (0) | 4/6 (66.7)  m: 100 | 0/6 (0) | 0/6 (0) |
| 2019 |  | 0/5 (0) | 1/5 (20) | 0/5 (0) | 0/5 (0) | 0/5 (0) | 5/5 (100)  m: 100 | 0/5 (0) |
| 2020 |  | 3/19 (15.8) | 0/19 (0) | 0/19 (0) | 0/19 (0) | 6/19 (31.6)  m: 100 | 4/19 (21.1)  m: 50 | 4/19 (21.1) |
| 2021 |  | 0/5 (0) | 0/5 (0) | 0/5 (0) | 0/5 (0) | 1/5 (20)  m: 100 | 0/5 (0) | 0/5 (0) |
| 2022 | 0/5 (0) | 1/8 (12.5) | 0/8 (0) | 0/8 (0) | 0/8 (0) | 2/8 (25)  m: 75 | 1/8 (12.5)  m: 50 | 1/8 (12.5) |
| 2023 | 0/4 (0) | 2/4 (50) | 0/4 (0) | 0/4 (0) | 0/4 (0) | 2/4 (50)  m: 75 | 1/4 (25)  m: 50 | 0/4 (0) |
| Results read as: positive samples/number of samples (proportion of positive samples %), m: median of EPG (eggs per gram of faeces), empty cells indicate not tested. *Other endoparasites were identified as *Capillaria* sp. and *Trichuris* sp. | | | | | | | | |

Table S8 – Overview of Konik horses parasite results, including number of samples tested per year and respective proportion of positive samples, and median eggs per gram of faeces (EPG).

| Year | Ectoparasites | *Eimeria* spp. | *Anoplocephala* spp. | *Paranoplocephala*  sp. | *Parascaris* sp. | *Oxyuris* sp. | Trichostrongylus/  Strongylus eggs | *S. westeri* | Other endoparasites |
| --- | --- | --- | --- | --- | --- | --- | --- | --- | --- |
| 1999 |  |  | 0/15 (0) | 0/15 (0) | 0/15 (0) | 0/15 (0) | 0/15 (0) | 0/15 (0) | 0/15 (0) |
| 2000 |  |  | 0/16 (0) | 0/16 (0) | 1/16 (6.2)  m: 350 | 0/16 (0) | 15/16 (93.8)  m: 1150 | 0/16 (0) | 0/16 (0) |
| 2001 |  |  | 0/15 (0) | 0/15 (0) | 0/15 (0) | 0/15 (0) | 15/15 (100)  m: 800 | 0/15 (0) | 0/15 (0) |
| 2002 |  |  | 0/10 (0) | 0/10 (0) | 0/10 (0) | 0/10 (0) | 10/10 (100)  m: 1125 | 0/10 (0) | 0/10 (0) |
| 2003 |  |  | 0/11 (0) | 0/11 (0) | 2/11 (18.2)  m: 200 | 0/11 (0) | 11/11 (100)  m: 3400 | 0/11 (0) | 0/11 (0) |
| 2004 |  |  | 0/15 (0) | 0/15 (0) | 1/15 (6.7)  m: 900 | 0/15 (0) | 14/15 (93.3)  m: 2425 | 1/15 (6.7)  m: 50 | 0/15 (0) |
| 2005 |  |  | 0/10 (0) | 0/10 (0) | 2/10 (20)  m: 50 | 0/10 (0) | 10/10 (100)  m: 1500 | 0/10 (0) | 0/10 (0) |
| 2007 |  |  | 0/17 (0) | 0/17 (0) | 2/17 (11.8)  m: 2650 | 0/17 (0) | 14/17 (82.4)  m: 1150 | 0/17 (0) | 0/17 (0) |
| 2011 |  | 0/6 (0) | 0/11 (0) | 0/11 (0) | 2/11 (18.2)  m: 100 | 0/11 (0) | 5/11 (45.5)  m: 100 | 0/11 (0) | 0/11 (0) |
| 2012 |  | 3/7 (42.9) | 0/11 (0) | 0/11 (0) | 0/11 (0) | 0/11 (0) | 7/11 (63.6)  m: 100 | 2/11 (18.2)  m: 50 | 0/11 (0) |
| 2013 |  | 0/3 (0) | 0/10 (0) | 0/10 (0) | 1/10 (10)  m: 200 | 0/10 (0) | 4/10 (40)  m: 75 | 1/10 (10)  m: 100 | 0/10 (0) |
| 2015 |  | 0/6 (0) | 0/10 (0) | 0/10 (0) | 1/10 (10)  m: 100 | 0/10 (0) | 5/10 (50)  m: 100 | 1/10 (10)  m: 100 | 0/10 (0) |
| 2018 |  |  | 0/3 (0) | 0/3 (0) | 0/3 (0) | 0/3 (0) | 2/3 (66.7)  m: 475 | 0/3 (0) | 0/3 (0) |
| 2019 |  | 0/2 (0) | 0/3 (0) | 0/3 (0) | 0/3 (0) | 0/3 (0) | 1/3 (33.3)  m: 2800 | 0/3 (0) | 0/3 (0) |
| 2020 |  | 1/7 (14.3) | 0/15 (0) | 0/15 (0) | 1/15 (6.7)  m: 300 | 0/15 (0) | 9/15 (60)  m: 450 | 0/15 (0) | 0/15 (0) |
| 2021 |  |  | 0/9 (0) | 0/9 (0) | 0/9 (0) | 0/9 (0) | 8/9 (88.9)  m: 675 | 0/9 (0) | 0/9 (0) |
| 2022 | 0/6 (0) |  | 0/8 (0) | 0/8 (0) | 0/8 (0) | 0/8 (0) | 6/8 (75)  m: 425 | 0/8 (0) | 0/8 (0) |
| 2023 | 0/9 (0) | 0/4 (0) | 0/9 (0) | 0/9 (0) | 0/9 (0) | 0/9 (0) | 8/9 (88.9)  m: 425 | 0/9 (0) | 0/9 (0) |
| Results read as: positive samples/number of samples (proportion of positive samples %), m: median of EPG (eggs per gram of faeces), empty cell indicate not tested. | | | | | | | | | |

## Probability of freedom from infection results

Table S9 – Results from probability of freedom from infection (PFFI) analysis in Heck cattle per pathogen per year based on a 25% design prevalence.

| **Pathogen** | **Year** | **n** | **Se** | **Sp** | **Prob. of H_0_** | **Prob. of H_a_** | **Confid. of freedom** | **Conclusion** |
| --- | --- | --- | --- | --- | --- | --- | --- | --- |
| ***B. abortus*** | 1997 | 1 | 20.7 | 100 | 0.948 | 1 | – | I.e. |
|  | 1998 | 15 | 20.7 | 100 | 0.451 | 1 | – | I.e. |
|  | 1999***** | 21 | 23.9 | 100 | 0.275 | 1 | – | I.e. |
|  | 2000***** | 22 | 25.4 | 99.9 | 0.230 | 1 | – | I.e. |
|  | 2001***** | 19 | 19 | 100 | 0.398 | 1 | – | I.e. |
|  | 2002***** | 18 | 20.1 | 100 | 0.396 | 1 | – | I.e. |
|  | 2003***** | 21 | 20.4 | 100 | 0.332 | 1 | – | I.e. |
|  | 2004***** | 17 | 20.9 | 100 | 0.402 | 1 | – | I.e. |
|  | 2005***** | 21 | 27 | 99.8 | 0.223 | 1 | – | I.e. |
|  | 2006***** | 18 | 18.7 | 100 | 0.423 | 1 | – | I.e. |
|  | 2007 | 20 | 17.6 | 100 | 0.407 | 1 | – | I.e. |
|  | 2008 | 13 | 81.7 | 98.7 | 0.042 | 1 | 0.958 | F |
|  | 2009 | 15 | 81.7 | 98.7 | 0.026 | 1 | 0.974 | F |
|  | 2010 | 21 | 81.7 | 98.7 | 0.006 | 1 | 0.994 | F |
|  | 2011 | 20 | 81.7 | 98.7 | 0.007 | 1 | 0.993 | F |
|  | 2012 | 20 | 81.7 | 98.7 | 0.007 | 1 | 0.993 | F |
|  | 2013***** | 24 | 73.7 | 98.9 | 0.005 | 1 | 0.995 | F |
|  | 2015***** | 9 | 80.6 | 99.1 | 0.119 | 1 | – | I.e. |
|  | 2016 | 1 | 81.7 | 98.7 | 0.787 | 1 | – | I.e. |
|  | 2018 | 21 | 81.7 | 98.7 | 0.005 | 1 | 0.995 | F |
|  | 2019 | 1 | 81.7 | 98.7 | 0.786 | 1 | – | I.e. |
|  | 2020***** | 6 | 81.7 | 98.9 | 0.239 | 1 | – | I.e. |
|  | 2021***** | 19 | 83.2 | 99 | 0.009 | 1 | 0.991 | F |
|  | 2022***** | 25 | 91.6 | 99.4 | 0.001 | 1 | 0.999 | F |
|  | 2023***** | 10 | 99.2 | 99.7 | 0.054 | 1 | – | I.e. |
| **BLV** | 1997 | 1 | 79.7 | 99 | 0.794 | 1 | – | I.e. |
|  | 1998 | 15 | 79.7 | 99 | 0.030 | 1 | 0.970 | F |
|  | 1999 | 20 | 79.7 | 99 | 0.009 | 1 | 0.991 | F |
|  | 2000 | 14 | 79.7 | 99 | 0.038 | 1 | 0.962 | F |
|  | 2001 | 18 | 79.7 | 99 | 0.015 | 1 | 0.985 | F |
|  | 2002 | 17 | 79.7 | 99 | 0.019 | 1 | 0.981 | F |
|  | 2003 | 21 | 79.7 | 99 | 0.007 | 1 | 0.993 | F |
|  | 2004 | 8 | 79.7 | 99 | 0.156 | 1 | – | I.e. |
|  | 2005***** | 20 | 82.7 | 99.1 | 0.008 | 1 | 0.992 | F |
|  | 2006 | 13 | 79.7 | 99 | 0.049 | 1 | 0.951 | F |
|  | 2007***** | 20 | 81.7 | 99.1 | 0.008 | 1 | 0.992 | F |
|  | 2008 | 15 | 79.7 | 99 | 0.030 | 1 | 0.970 | F |
|  | 2009 | 14 | 79.7 | 99 | 0.038 | 1 | 0.962 | F |
|  | 2010 | 21 | 99.6 | 99.6 | 0.002 | 1 | 0.998 | F |
|  | 2011 | 20 | 99.6 | 99.6 | 0.003 | 1 | 0.997 | F |
|  | 2012 | 18 | 99.6 | 99.6 | 0.004 | 1 | 0.996 | F |
|  | 2013 | 25 | 99.6 | 99.6 | 0.000 | 1 | 1.000 | F |
|  | 2015 | 9 | 99.6 | 99.6 | 0.070 | 1 | – | I.e. |
|  | 2016 | 1 | 99.6 | 99.6 | 0.750 | 1 | – | I.e. |
|  | 2017 | 2 | 99.6 | 99.6 | 0.558 | 1 | – | I.e. |
|  | 2018 | 21 | 99.6 | 99.6 | 0.002 | 1 | 0.998 | F |
|  | 2019 | 1 | 99.6 | 99.6 | 0.748 | 1 | – | I.e. |
|  | 2020 | 6 | 99.6 | 99.6 | 0.175 | 1 | – | I.e. |
|  | 2021 | 19 | 99.6 | 99.6 | 0.003 | 1 | 0.997 | F |
|  | 2022 | 26 | 99.6 | 99.6 | 0.000 | 1 | 1.000 | F |
|  | 2023 | 10 | 99.6 | 99.6 | 0.053 | 1 | – | I.e. |
| **bTB** | 1997 | 1 | 33.9 | 100 | 0.915 | 1 | – | I.e. |
|  | 1998 | 15 | 33.9 | 100 | 0.265 | 1 | – | I.e. |
|  | 1999***** | 18 | 28.5 | 100 | 0.264 | 1 | – | I.e. |
|  | 2000***** | 20 | 31.6 | 100 | 0.191 | 1 | – | I.e. |
|  | 2001 | 15 | 26.5 | 100 | 0.358 | 1 | – | I.e. |
|  | 2002***** | 17 | 56.8 | 100 | 0.073 | 1 | – | I.e. |
|  | 2003 | 21 | 78.1 | 100 | 0.010 | 1 | 0.990 | F |
|  | 2004 | 17 | 78.1 | 100 | 0.024 | 1 | 0.976 | F |
|  | 2005 | 18 | 78.1 | 100 | 0.019 | 1 | 0.981 | F |
|  | 2006 | 18 | 78.1 | 100 | 0.019 | 1 | 0.981 | F |
|  | 2007 | 31 | 78.1 | 100 | 0.001 | 1 | 0.999 | F |
|  | 2008 | 10 | 78.1 | 100 | 0.112 | 1 | – | I.e. |
|  | 2009 | 6 | 78.1 | 100 | 0.273 | 1 | – | I.e. |
|  | 2010 | 18 | 78.1 | 100 | 0.019 | 1 | 0.981 | F |
|  | 2011 | 5 | 78.1 | 100 | 0.340 | 1 | – | I.e. |
|  | 2012 | 7 | 78.1 | 100 | 0.215 | 1 | – | I.e. |
|  | 2013 | 10 | 78.1 | 100 | 0.113 | 1 | – | I.e. |
|  | 2015 | 9 | 78.1 | 100 | 0.138 | 1 | – | I.e. |
|  | 2017 | 1 | 78.1 | 100 | 0.805 | 1 | – | I.e. |
|  | 2018 | 6 | 78.1 | 100 | 0.275 | 1 | – | I.e. |
|  | 2019 | 1 | 78.1 | 100 | 0.805 | 1 | – | I.e. |
|  | 2020 | 6 | 78.1 | 100 | 0.272 | 1 | – | I.e. |
|  | 2021 | 12 | 78.1 | 100 | 0.072 | 1 | – | I.e. |
|  | 2022 | 10 | 78.1 | 100 | 0.111 | 1 | – | I.e. |
|  | 2023 | 5 | 78.1 | 100 | 0.337 | 1 | – | I.e. |
| **FMDV** | 2012 | 5 | 98.3 | 99.2 | 0.231 | 1 | – | I.e. |
|  | 2013 | 13 | 98.3 | 99.2 | 0.021 | 1 | 0.979 | F |
| ***L.* Hardjo** | 1997 | 1 | 97.6 | 99.6 | 0.754 | 1 | – | I.e. |
|  | 1998 | 15 | 97.6 | 99.6 | 0.013 | 1 | 0.987 | F |
|  | 1999 | 20 | 97.6 | 99.6 | 0.003 | 1 | 0.997 | F |
|  | 2000 | 19 | 97.6 | 99.6 | 0.004 | 1 | 0.996 | F |
|  | 2001 | 17 | 97.6 | 99.6 | 0.008 | 1 | 0.992 | F |
|  | 2002 | 17 | 97.6 | 99.6 | 0.008 | 1 | 0.992 | F |
|  | 2003 | 21 | 97.6 | 99.6 | 0.002 | 1 | 0.998 | F |
|  | 2004 | 8 | 97.6 | 99.6 | 0.102 | 1 | – | I.e. |
|  | 2005 | 20 | 97.6 | 99.6 | 0.003 | 1 | 0.997 | F |
|  | 2006 | 13 | 97.6 | 99.6 | 0.024 | 1 | 0.976 | F |
|  | 2007 | 20 | 97.6 | 99.6 | 0.003 | 1 | 0.997 | F |
|  | 2008 | 16 | 97.6 | 99.6 | 0.010 | 1 | 0.990 | F |
|  | 2009 | 17 | 97.6 | 99.6 | 0.007 | 1 | 0.993 | F |
|  | 2010 | 21 | 97.6 | 99.6 | 0.002 | 1 | 0.998 | F |
|  | 2011 | 20 | 97.6 | 99.6 | 0.003 | 1 | 0.997 | F |
|  | 2012***** | 15 | 93.5 | 99.3 | 0.014 | 1 | 0.986 | F |
|  | 2013***** | 24 | 96.3 | 99.5 | 0.001 | 1 | 0.999 | F |
|  | 2015 | 9 | 97.6 | 99.6 | 0.074 | 1 | – | I.e. |
|  | 2016 | 1 | 97.6 | 99.6 | 0.755 | 1 | – | I.e. |
|  | 2017 | 1 | 97.6 | 99.6 | 0.753 | 1 | – | I.e. |
|  | 2018 | 21 | 97.6 | 99.6 | 0.002 | 1 | 0.998 | F |
|  | 2019 | 1 | 97.6 | 99.6 | 0.753 | 1 | – | I.e. |
|  | 2020 | 6 | 97.6 | 99.6 | 0.182 | 1 | – | I.e. |
|  | 2021 | 19 | 97.6 | 99.6 | 0.004 | 1 | 0.996 | F |
|  | 2022 | 25 | 97.6 | 99.6 | 0.001 | 1 | 0.999 | F |
|  | 2023 | 10 | 97.6 | 99.6 | 0.057 | 1 | – | I.e. |
| ***S*. Typhimurium** | 2000 | 1 | 73.8 | 93.2 | 0.764 | 1 | – | I.e. |
|  | 2001 | 2 | 73.8 | 93.2 | 0.585 | 1 | – | I.e. |
|  | 2002 | 10 | 73.8 | 93.2 | 0.068 | 1 | – | I.e. |
|  | 2003 | 19 | 73.8 | 93.2 | 0.006 | 1 | 0.994 | F |
|  | 2004 | 6 | 73.8 | 93.2 | 0.199 | 1 | – | I.e. |
|  | 2005 | 17 | 73.8 | 93.2 | 0.010 | 1 | 0.990 | F |
|  | 2006 | 13 | 73.8 | 93.2 | 0.030 | 1 | 0.970 | F |
|  | 2007 | 20 | 73.8 | 93.2 | 0.004 | 1 | 0.996 | F |
|  | 2008 | 11 | 73.8 | 93.2 | 0.051 | 1 | – | I.e. |
|  | 2009 | 15 | 73.8 | 93.2 | 0.018 | 1 | 0.982 | F |
|  | 2010 | 21 | 73.8 | 93.2 | 0.003 | 1 | 0.997 | F |
|  | 2011 | 10 | 73.8 | 93.2 | 0.068 | 1 | – | I.e. |
|  | 2012 | 15 | 73.8 | 93.2 | 0.017 | 1 | 0.983 | F |
|  | 2013 | 18 | 73.8 | 93.2 | 0.007 | 1 | 0.993 | F |
|  | 2015 | 9 | 73.8 | 93.2 | 0.088 | 1 | – | I.e. |
|  | 2016 | 1 | 73.8 | 93.2 | 0.766 | 1 | – | I.e. |
|  | 2017 | 2 | 73.8 | 93.2 | 0.584 | 1 | – | I.e. |
|  | 2018 | 21 | 73.8 | 93.2 | 0.003 | 1 | 0.997 | F |
|  | 2019 | 1 | 73.8 | 93.2 | 0.764 | 1 | – | I.e. |
|  | 2020 | 6 | 73.8 | 93.2 | 0.200 | 1 | – | I.e. |
|  | 2021 | 18 | 73.8 | 93.2 | 0.008 | 1 | 0.992 | F |
|  | 2022 | 24 | 73.8 | 93.2 | 0.001 | 1 | 0.999 | F |
|  | 2023 | 9 | 73.8 | 93.2 | 0.088 | 1 | – | I.e. |
| **F** free from infection, **I.e.** insufficient evidence due to small sample size (taking into account different Se/Sp)  *****weighted estimates | | | | | | | | |

Table S10 – Results from probability of freedom from infection (PFFI) analysis in red deer per pathogen per year based on a 25% design prevalence.

| **Pathogen** | **Year** | **n** | **Se** | **Sp** | **Prob. of H_0_** | **Prob. of H_a_** | **Confid. of freedom** | **Conclusion** |
| --- | --- | --- | --- | --- | --- | --- | --- | --- |
| **B. abortus** | 2000 | 2 | 21.6 | 100 | 0.895 | 1 | – | I.e. |
|  | 2001***** | 2 | 19.3 | 100 | 0.906 | 1 | – | I.e. |
|  | 2002 | 1 | 21.6 | 100 | 0.946 | 1 | – | I.e. |
|  | 2003***** | 2 | 19.6 | 100 | 0.904 | 1 | – | I.e. |
|  | 2005 | 2 | 81.7 | 98.7 | 0.618 | 1 | – | I.e. |
|  | 2007 | 8 | 81.7 | 98.7 | 0.146 | 1 | – | I.e. |
|  | 2011 | 14 | 81.7 | 98.7 | 0.034 | 1 | 0.966 | F |
|  | 2012 | 9 | 81.7 | 98.7 | 0.114 | 1 | – | I.e. |
|  | 2013 | 18 | 81.7 | 98.7 | 0.013 | 1 | 0.987 | F |
|  | 2015***** | 5 | 81 | 99 | 0.307 | 1 | – | I.e. |
|  | 2016 | 5 | 81.7 | 98.7 | 0.300 | 1 | – | I.e. |
|  | 2017 | 11 | 81.7 | 98.7 | 0.071 | 1 | – | I.e. |
|  | 2018***** | 16 | 83.5 | 98.8 | 0.020 | 1 | 0.980 | F |
|  | 2019 | 14 | 81.7 | 98.7 | 0.034 | 1 | 0.966 | F |
|  | 2020 | 19 | 81.7 | 98.7 | 0.010 | 1 | 0.990 | F |
|  | 2021***** | 15 | 87.2 | 99.1 | 0.022 | 1 | 0.978 | F |
|  | 2022***** | 8 | 91.9 | 99.4 | 0.118 | 1 | – | I.e. |
|  | 2023***** | 4 | 99 | 99.7 | 0.316 | 1 | – | I.e. |
| **BLV** | 2002 | 1 | 79.7 | 99 | 0.794 | 1 | – | I.e. |
|  | 2003 | 2 | 79.7 | 99 | 0.629 | 1 | – | I.e. |
|  | 2022 | 1 | 99.6 | 99.6 | 0.748 | 1 | – | I.e. |
| **bTB** | 2000***** | 2 | 52.3 | 100 | 0.756 | 1 | – | I.e. |
|  | 2001***** | 2 | 52.3 | 100 | 0.757 | 1 | – | I.e. |
|  | 2002 | 1 | 78.1 | 100 | 0.805 | 1 | – | I.e. |
|  | 2003 | 2 | 78.1 | 100 | 0.648 | 1 | – | I.e. |
|  | 2011 | 2 | 78.1 | 100 | 0.648 | 1 | – | I.e. |
|  | 2012***** | 3 | 78.1 | 100 | 0.522 | 1 | – | I.e. |
|  | 2013 | 11 | 78.1 | 100 | 0.092 | 1 | – | I.e. |
| **BVDV ab** | 2000 | 1 | 98 | 99 | 0.748 | 1 | – | I.e. |
|  | 2001 | 2 | 98 | 99 | 0.560 | 1 | – | I.e. |
|  | 2002 | 1 | 98 | 99 | 0.748 | 1 | – | I.e. |
|  | 2003 | 2 | 98 | 99 | 0.559 | 1 | – | I.e. |
|  | 2005 | 2 | 98 | 99 | 0.559 | 1 | – | I.e. |
|  | 2007 | 8 | 98 | 99 | 0.097 | 1 | – | I.e. |
|  | 2011 | 7 | 98 | 99 | 0.130 | 1 | – | I.e. |
|  | 2012 | 10 | 98 | 99 | 0.054 | 1 | – | I.e. |
|  | 2013 | 13 | 98 | 99 | 0.023 | 1 | 0.977 | F |
|  | 2015 | 6 | 98 | 99 | 0.174 | 1 | – | I.e. |
|  | 2016 | 5 | 98 | 99 | 0.233 | 1 | – | I.e. |
|  | 2017 | 13 | 98 | 99 | 0.023 | 1 | 0.977 | F |
|  | 2018 | 16 | 98 | 99 | 0.009 | 1 | 0.991 | F |
|  | 2019 | 15 | 98 | 99 | 0.012 | 1 | 0.988 | F |
|  | 2020 | 21 | 98 | 99 | 0.002 | 1 | 0.998 | F |
|  | 2021 | 15 | 98 | 99 | 0.013 | 1 | 0.987 | F |
|  | 2022 | 11 | 98 | 99 | 0.040 | 1 | 0.960 | F |
|  | 2023 | 6 | 98 | 99 | 0.173 | 1 | – | I.e. |
| **BVDV ag** | 2000 | 2 | 99 | 99.5 | 0.561 | 1 | – | I.e. |
|  | 2001 | 2 | 99 | 99.5 | 0.562 | 1 | – | I.e. |
|  | 2003 | 1 | 99 | 99.5 | 0.749 | 1 | – | I.e. |
|  | 2005 | 2 | 99 | 99.5 | 0.561 | 1 | – | I.e. |
|  | 2007 | 8 | 99 | 99.5 | 0.099 | 1 | – | I.e. |
|  | 2011 | 7 | 99 | 99.5 | 0.132 | 1 | – | I.e. |
|  | 2012 | 10 | 99 | 99.5 | 0.055 | 1 | – | I.e. |
|  | 2013 | 19 | 99 | 99.5 | 0.004 | 1 | 0.996 | F |
|  | 2015 | 6 | 99 | 99.5 | 0.176 | 1 | – | I.e. |
|  | 2016 | 5 | 99 | 99.5 | 0.235 | 1 | – | I.e. |
|  | 2017 | 13 | 99 | 99.5 | 0.023 | 1 | 0.977 | F |
|  | 2018 | 16 | 99 | 99.5 | 0.010 | 1 | 0.990 | F |
|  | 2019 | 15 | 99 | 99.5 | 0.013 | 1 | 0.987 | F |
|  | 2020 | 21 | 99 | 99.5 | 0.002 | 1 | 0.998 | F |
|  | 2021 | 15 | 99 | 99.5 | 0.013 | 1 | 0.987 | F |
|  | 2022 | 11 | 99 | 99.5 | 0.041 | 1 | 0.959 | F |
|  | 2023 | 5 | 99 | 99.5 | 0.234 | 1 | – | I.e. |
| ***C. burnetii*** | 2011***** | 14 | 90.7 | 98.6 | 0.022 | 1 | 0.978 | F |
|  | 2012***** | 8 | 91.6 | 99.2 | 0.118 | 1 | – | I.e. |
|  | 2013***** | 18 | 91.3 | 99 | 0.008 | 1 | 0.992 | F |
|  | 2016 | 5 | 92.5 | 100 | 0.269 | 1 | – | I.e. |
|  | 2017 | 12 | 92.5 | 100 | 0.042 | 1 | 0.958 | F |
|  | 2018 | 9 | 92.5 | 100 | 0.094 | 1 | – | I.e. |
| ***L.* Hardjo** | 2000 | 1 | 97.6 | 99.6 | 0.754 | 1 | – | I.e. |
|  | 2001 | 2 | 97.6 | 99.6 | 0.569 | 1 | – | I.e. |
|  | 2002 | 1 | 97.6 | 99.6 | 0.754 | 1 | – | I.e. |
|  | 2003 | 2 | 97.6 | 99.6 | 0.567 | 1 | – | I.e. |
|  | 2011***** | 14 | 88.9 | 98.9 | 0.025 | 1 | 0.975 | F |
|  | 2012***** | 7 | 80.1 | 98.1 | 0.185 | 1 | – | I.e. |
|  | 2013***** | 22 | 83.7 | 98.4 | 0.004 | 1 | 0.996 | F |
|  | 2016 | 4 | 67 | 97 | 0.431 | 1 | – | I.e. |
|  | 2017***** | 12 | 95 | 99.4 | 0.036 | 1 | 0.964 | F |
|  | 2018 | 11 | 67 | 97 | 0.098 | 1 | – | I.e. |
|  | 2019 | 8 | 67 | 97 | 0.185 | 1 | – | I.e. |
|  | 2020 | 2 | 67 | 97 | 0.656 | 1 | – | I.e. |
|  | 2021 | 4 | 67 | 97 | 0.431 | 1 | – | I.e. |
|  | 2022***** | 3 | 77.2 | 97.9 | 0.495 | 1 | – | I.e. |
|  | 2023 | 4 | 67 | 97 | 0.430 | 1 | – | I.e. |
| ***Salmonella* spp.** | 2000 | 1 | 20 | 100 | 0.950 | 1 | – | I.e. |
|  | 2001 | 2 | 20 | 100 | 0.903 | 1 | – | I.e. |
|  | 2002 | 1 | 20 | 100 | 0.950 | 1 | – | I.e. |
|  | 2003 | 2 | 20 | 100 | 0.903 | 1 | – | I.e. |
|  | 2005 | 2 | 20 | 100 | 0.903 | 1 | – | I.e. |
|  | 2007 | 8 | 20 | 100 | 0.664 | 1 | – | I.e. |
|  | 2011 | 10 | 76 | 100 | 0.121 | 1 | – | I.e. |
|  | 2012 | 3 | 70 | 100 | 0.562 | 1 | – | I.e. |
|  | 2013 | 12 | 70 | 100 | 0.099 | 1 | – | I.e. |
|  | 2015 | 6 | 70 | 100 | 0.315 | 1 | – | I.e. |
|  | 2017 | 1 | 70 | 100 | 0.825 | 1 | – | I.e. |
|  | 2018 | 6 | 70 | 100 | 0.316 | 1 | – | I.e. |
|  | 2019 | 5 | 70 | 100 | 0.382 | 1 | – | I.e. |
|  | 2020 | 17 | 70 | 100 | 0.038 | 1 | 0.962 | F |
|  | 2021 | 5 | 70 | 100 | 0.382 | 1 | – | I.e. |
|  | 2022***** | 8 | 45 | 100 | 0.385 | 1 | – | I.e. |
|  | 2023***** | 4 | 45 | 100 | 0.620 | 1 | – | I.e. |
| **F** free from infection, **I.e.** insufficient evidence due to small sample size (taking into account different Se/Sp)  *****weighted estimates | | | | | | | | |

Table S11 – Results from probability of freedom from infection (PFFI) analysis in Konik horses per pathogen per year based on a 25% design prevalence.

| **Pathogen** | **Year** | **n** | **Se** | **Sp** | **Prob. of H_0_** | **Prob. of H_a_** | **Confid. of freedom** | **Conclusion** |
| --- | --- | --- | --- | --- | --- | --- | --- | --- |
| **EIAV** | 2013 | 9 | 98.8 | 100 | 0.077 | 1 | – | I.e. |
|  | 2016 | 8 | 98.8 | 100 | 0.103 | 1 | – | I.e. |
|  | 2018 | 9 | 98.8 | 100 | 0.076 | 1 | – | I.e. |
| **EIV** | 2013 | 2 | 98.5 | 95.4 | 0.518 | 1 | – | I.e. |
| **EIV Am** | 2012 | 6 | 95 | 100 | 0.195 | 1 | – | I.e. |
|  | 2016 | 8 | 95 | 100 | 0.114 | 1 | – | I.e. |
| **EIV Eu** | 2012 | 6 | 95 | 100 | 0.195 | 1 | – | I.e. |
|  | 2016 | 8 | 95 | 100 | 0.114 | 1 | – | I.e. |
| **WNV** | 2012 | 6 | 96.5 | 100 | 0.190 | 1 | – | I.e. |
|  | 2013 | 9 | 96.5 | 100 | 0.083 | 1 | – | I.e. |
|  | 2016 | 8 | 96.5 | 100 | 0.109 | 1 | – | I.e. |
| **Am** = American lineage; **Eu** = European lineage  **F** = free from infection; **I.e.** = insufficient evidence due to small sample size (taking into account different Se/Sp) | | | | | | | | |
